# Pseudorabies virus induces natural killer cell depletion by GSDMD-mediated inflammation and pyroptosis to promote infection and lung injury

**DOI:** 10.1128/jvi.00415-25

**Published:** 2025-07-24

**Authors:** Jing Chen, Guangming Zhao, Yi Yang, Yinhui Li, Yu Song, Daxia Li, Qian Du, Dewen Tong, Yong Huang

**Affiliations:** 1 College of Veterinary Medicine, Northwest A&F University 718173, Yangling, China; 2 Shaanxi Provincial Animal Disease Prevention and Control Center, Xi’an, China; 3 Engineering Research Center of Efficient New Vaccines for Animals, Ministry of Education of the People’s Republic of China 12543, Yangling, China; 4 Key Laboratory of Ruminant Disease Prevention and Control (West), Ministry of Agriculture and Rural Affairs 12654 , Yangling, China; 5 Engineering Research Center of Efficient New Vaccines for Animals, Universities of Shaanxi Province, Yangling, China; Lerner Research Institute, Cleveland Clinic, Cleveland, Ohio, USA

**Keywords:** pseudorabies virus, lung injury, NK cell

## Abstract

Pseudorabies virus (PRV) infection induces a hyperinflammatory response to promote inflammatory injury in multiple tissues. However, the relative mechanism remains elusive. Herein, we first confirmed that PRV infection could trigger pyroptosis to cause lung injury, evidenced by the increased release of pro-inflammatory cytokines and lactate dehydrogenase (LDH) in lung tissue of infected piglets and mice. Subsequently, we observed that PRV-induced lung lesions and mortality were improved in *Gsdmd^−/−^* mice rather than in *Ripk3^−/−^* mice. The number of NK cells was markedly higher in *Gsdmd^−/−^* mice than in WT mice. Based on this finding, refilling NK cells could significantly alleviate the replication of PRV in the lungs of mice and alleviate lung injury caused by PRV infection, whereas depletion of NK cells aggravated PRV infection and led to severe lung lesions. Notably, gasdermin D (GSDMD) deficiency could reduce the production of tumor necrosis factor-α (TNF-α) positive macrophages and the depletion of NK cells induced by PRV infection to alleviate the pathogenicity of PRV, while TNF-α neutralizing antibody could also reduce the PRV-induced NK cell depletion to alleviate the pathogenicity of PRV. Ultimately, we found that necrosulfonamide (NSA) showed a good protective effect on PRV-infected mice and had good anti-PRV activity *in vivo*. Collectively, our findings illuminate a new regulatory mechanism by which PRV infection induces NK cell depletion to weaken the antiviral ability of the host to promote virus replication and activate GSDMD-mediated pyroptosis in lung tissue, leading to severer lung injury, and NSA is expected to be a candidate agent for the prevention and control of PRV infection.

**IMPORTANCE:**

Necroptosis and pyroptosis are the most common regulated necrotic cell death pathways in various pathogenic infection-induced tissue damage. Herein, we found that gasdermin D (GSDMD)-mediated pyroptosis is a critical cell death pathway in pseudorabies virus (PRV)-induced lung inflammatory injury rather than receptor-interacting protein kinase 3 (RIPK3)-mediated necroptosis. PRV infection induces NK cell depletion to weaken the antiviral ability of the host to promote viral replication. GSDMD deficiency can reduce the depletion of NK cells induced by PRV infection by reducing the production of tumor necrosis factor-α (TNF-α)-positive macrophages, thereby attenuating lung tissue lesions in PRV-infected mice. Whereas the use of necrosulfonamide (NSA) showed a good protective effect on PRV-infected mice. This study reveals that PRV infection can excessively activate macrophages to secrete more TNF-α to induce NK cell depletion to facilitate PRV infection and aggravate viral pathogenicity, and clarifies the roles of GSDMD in modulating macrophage activity and NK cell death, and also provides an effective inhibitor for use against PRV infection.

## INTRODUCTION

Pseudorabies, also known as Aujeszky’s disease (1), was first described in America as early as 1813 and has spread nearly globally since the early 1980s. Its etiological agent is pseudorabies virus (PRV), also called Aujeszky’s disease virus or *Suid alphaherpesvirus* 1, which is an enveloped, linear double-stranded DNA herpes virus with 143 kb and can encode more than 70 proteins belonging to the *Varicellovirus* genus of subfamily *Alphaherpesvirinae* in the family *Herpesviridae* (2). Many domestic and wild animals other than pigs are susceptible to PRV-RJ infection, such as cattle, sheep, dogs, cats, rodents, and other vertebrates (3). PRV infection causes lung tissue lesions (4), and infected cells have an inflammatory response (5, 6), with the secretion of interleukin-1β (IL-1β) and tumor necrosis factor-α (TNF-α) (7).

Necroptosis and pyroptosis are the most common regulated necrotic cell death pathways in various pathogenic infection-induced tissue damage (8). On one hand, receptor-interacting protein kinase 3 (RIPK3)-mediated necroptosis could be triggered by biological stimuli, including TNF and interferons (IFNs) (9). On the other hand, pyroptosis occurring upon activation of proinflammatory caspases and their subsequent cleavage of gasdermin D (GSDMD) is a primary cellular response to various cellular insults, including pathogen-associated molecular patterns, DAMPs, altered levels of host metabolites, and environmental irritants (10). It is known that pyroptosis is involved in the immune response in various inflammatory cytokines and intracellular contents. However, there are few studies on whether GSDMD-mediated pyroptosis and RIPK3-mediated necroptosis of cells are involved in PRV-induced lung injury.

A kind of pathological manifestation in lung injury is inflammatory cell infiltration. Inflammatory cell is a type of immune cell in the animal’s body, which includes neutrophils, eosinophils, mononuclear macrophages, lymphocytes, and plasma cells (11). These cells play an important role in the body’s resistance to invading bacteria and microorganisms. For example, macrophages can engulf and destroy the injured tissue, which can help start the rehabilitation process (12). While they play a critical role at the site of injury, once the job is done, they need to be withdrawn as soon as possible to end the inflammatory response and make way for the regeneration process. The continued presence of macrophages is detrimental to tissue recovery (12). NK cells are innate lymphocytes with an array of functional competences, including anti-viral and anti-graft- vs -host disease potential (13). A variety of activating and inhibitory receptors are expressed on the NK-cell surface, and the balance and spatial-temporal integration of signals from these receptors determine the NK-cell effector functions. As NK-cell functions are regulated by the integration of activating and inhibitory signals from cell surface receptors, reduced or weakened signals from activating receptors will lead to domination by inhibitory signals and decreased NK-cell functionality (14–16). Functionally impaired NK cells exhibit a decreased expression of the transient degranulation marker lysosomal-associated membrane glycoprotein-1, a decreased production of the cytokine interferon-γ and TNF-α, and a decreased production of the cytotoxic secretions of perforin and granzymes (17). GSDMD can mediate pyroptosis of pulmonary macrophages caused by *Streptococcus pneumoniae* infection (18). When GSDMD is inhibited, it can effectively reduce the death of macrophages and reduce the damage of pneumonia (18). In lung cancer, GSDMD facilitates cytotoxic T lymphocytes to kill cancer cells (19). However, it has not been reported that the effect of GSDMD on immune cells in lung injury induced by PRV infection.

Swine, including domestic pigs and wild boar, represent the natural host of PRV, where the virus infection causes lung injury and inflammatory imbalance. Yet, how PRV infection causes systemic inflammatory responses and inflammatory damages is still poorly understood. This study attempts to reveal the mechanism of inflammatory damage caused by PRV infection from the perspective of immune regulation. In this study, we first constructed a model of lung injury caused by PRV infection and found that PRV infection activated GSDMD-mediated pyroptosis, but not the influence of PRV on RIPK3. Subsequently, according to the single-cell sequencing, the inflammatory cell populations of the lung in WT mice and *Gsdmd^−/−^* mice with PRV infection were identified. At the same time, we identified key cell populations in PRV-infected WT mice compared with PRV-infected *Gsdmd^−/−^* mice and then verified their role and mechanism in the pathogenesis of PRV infection. Finally, we investigated the role of necrosulfonamide (NSA) in the pathogenesis of PRV infection. These results provide new insights into the roles of lung injury during PRV infection and lay a theoretical foundation for the subsequent study of the specific mechanism, providing a target for clinical prevention and treatment of Pseudorabies.

## RESULTS

### PRV infection causes lung injury and activates GSDMD-mediated pyroptosis in piglets

**Fig 1:**
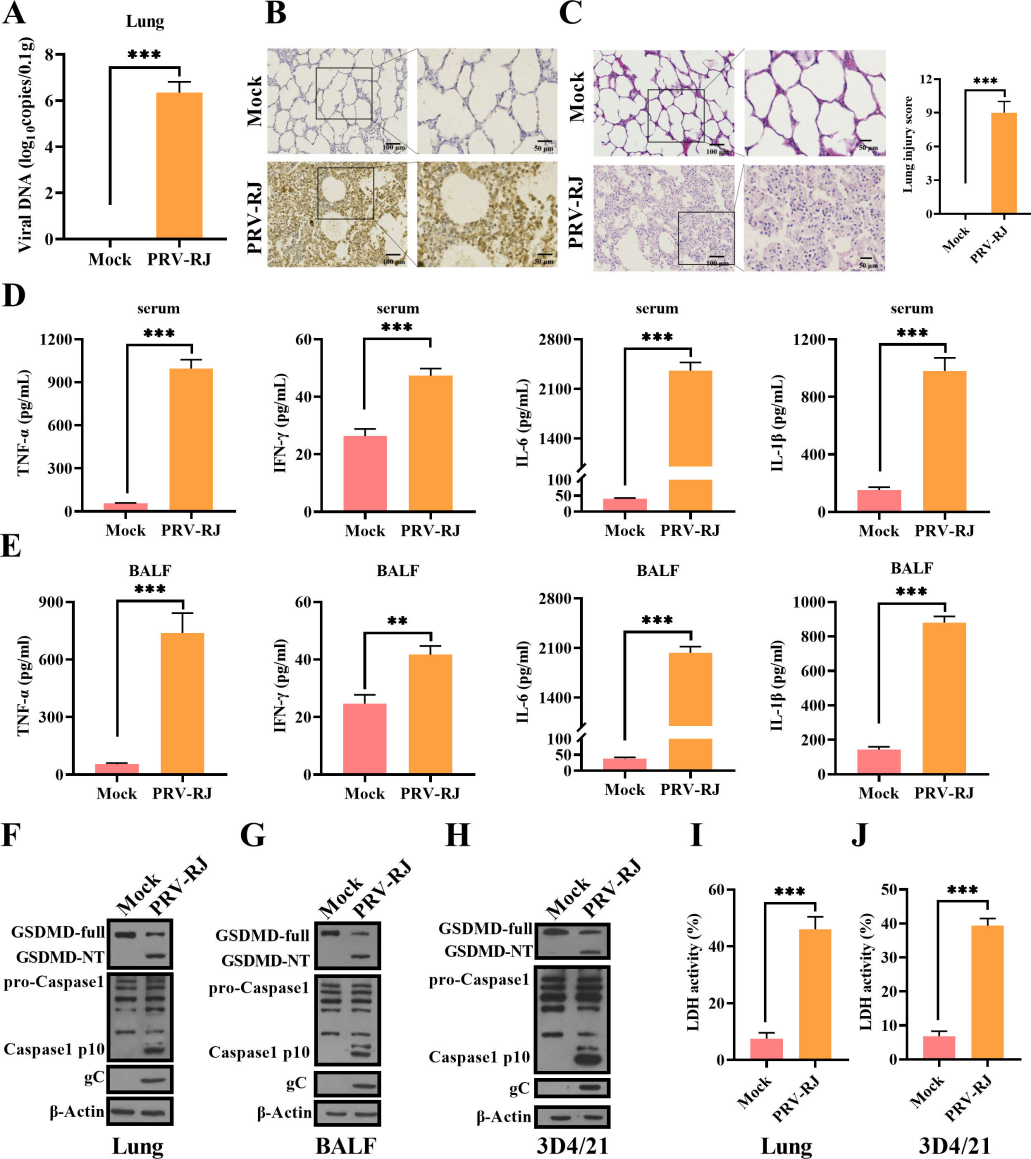
PRV infection causes lung injury and activates pyroptosis in piglets. Six-week-old piglets (n = 6 per group) were inoculated subcutaneously with PBS or 4 × 10^5^ TCID_50_ PRV-RJ for 4 days. (**A**) Viral copies of lung tissues were detected by quantitative PCR (qPCR). (**B**) Immunohistochemistry detection of PRV-RJ-gC in piglets. (**C**) Pathological lesions of piglets (hematoxylin and eosin [H&E] staining) from the lung were shown, and histological scoring of lung injury. (**D and E**) Detection of proinflammatory cytokines (TNF-α, IFN-γ, IL-6, and IL-1β) in serum (**D**) and BALF (**E**) by enzyme-linked immunosorbent assay (ELISA). (**F through H**) The protein levels of GSDMD-full, GSDMD-NT, pro-Caspase-1, p10, and PRV-RJ-gC in lung tissues (**F**), BALF (**G**), and 3D4/21 cells (**H**) were analyzed by western blot. (**I**) The LDH assay in lung tissues was measured. (**J**) The LDH assay in 3D/421 cells was measured. **, *P* < 0.01; ***, *P* < 0.001.

PRV is characterized by respiratory distress in growing pigs. Previous studies have shown that PRV infection causes systemic inflammatory responses and inflammatory damage in infected animals, which are associated with the activation of the inflammasome and pyroptosis in infected tissues (20, 21). To further systematically explore the mechanism of lung injury caused by PRV infection, we used 4 × 10^5^ TCID_50_ PRV-RJ strain to infect 6-week-old pigs via nasal drops. Four days after infection, PRV-RJ was detectable in the lung tissues of the PRV-RJ-infected piglets (Fig. 1A and B), and the microscopic lesions of typical interstitial pneumonia characterized by narrowed alveolar spaces and thickened alveolar septa with inflammatory cell infiltration were observed in the PRV-RJ-infected lung tissue (Fig. 1C). Histopathological damage even led to partial disappearance of lung structure in PRV-RJ-infected piglets (Fig. 1C). In the serum and bronchoalveolar lavage fluid (BALF) of PRV-infected piglets, the release levels of TNF-α, IFN-γ, IL-6, and IL-1β were markedly increased compared with those of the mock-infected group, especially the concentration of IL-1β in the serum and BALF, which was significantly increased in PRV-RJ-infected piglets (Fig. 1D and E). Next, we tested whether PRV infection can activate the inflammasome to induce GSDMD cleavage in infected lung tissues. As expected, the cleavage fragments of Caspase-1 (p10) and the N-terminal cleavage fragments of GSDMD (GSDMD-NT) were detected in PRV-RJ-infected lung tissues (Fig. 1F). Meanwhile, in the cells washed out from the alveolar lavage of PRV-infected piglets, the cleavage of Caspase-1 and the cleavage of GSDMD were also detected (Fig. 1G). In 3D4/21 cells, the cleavage of Caspase-1 and the cleavage of GSDMD were also detected in PRV-infected cells (Fig. 1H). As a result, the release level of lactate dehydrogenase (LDH) in PRV-infected lung tissues was significantly higher than that in mock-infected lung tissues, as well as in PRV-infected 3D4/21 cells (Fig. 1I and J). These results suggested that PRV infection could activate the inflammasome to induce GSDMD cleavage in lung tissue cells and the infiltration of inflammatory cells, which might promote the occurrence of pyroptosis in the lung tissue cells and inflammatory cells and cause lung tissue injury.

### PRV infection causes lung injury and activates GSDMD-mediated pyroptosis in mice

**Fig 2:**
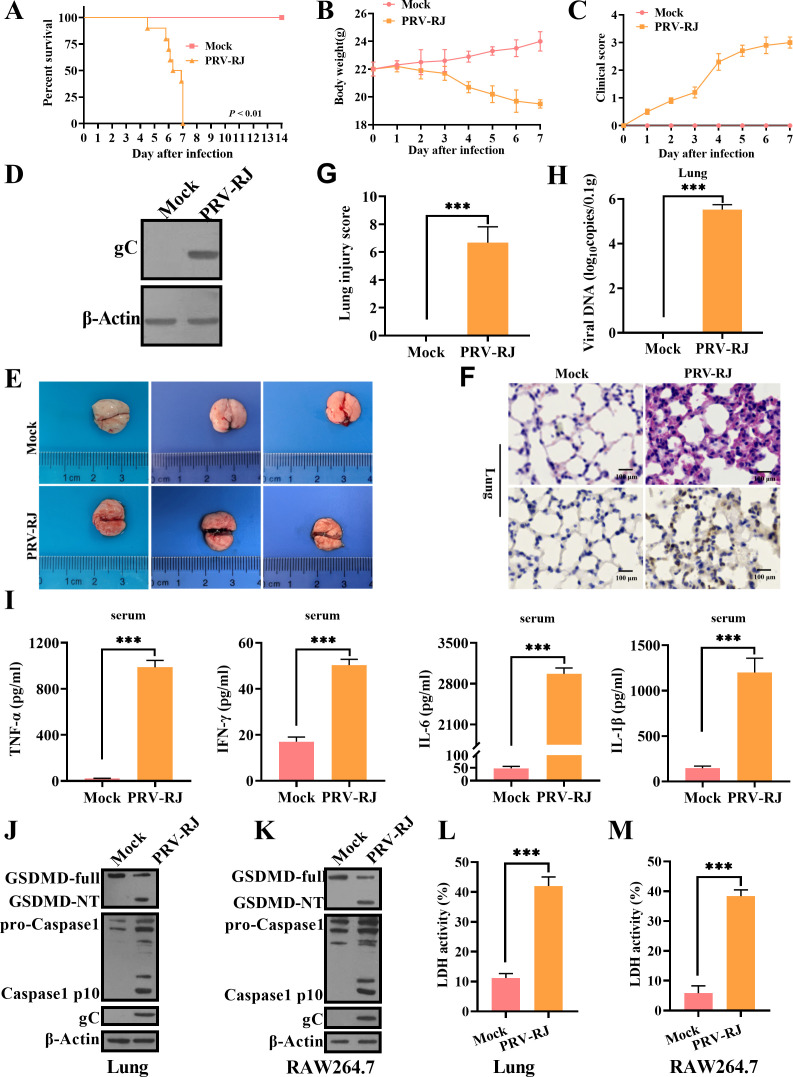
PRV infection causes lung injury and activates pyroptosis in mice. (A–C) WT mice (n = 10 per group) were inoculated intraperitoneally with PBS or 2 × 10^3^ TCID_50_ PRV-RJ for 14 days. Survival rate (**A**), body weight (**B**), and clinic score (**C**) of WT mice infected with PRV-RJ. (**D through J and L**) WT mice (n = 6 per group) were inoculated intraperitoneally with PBS or 2 × 10^3^ TCID_50_ PRV-RJ, and then all mice were sacrificed on the fourth day. (**D**) The protein level of PRV-gC was analyzed by western blot from lung tissues. (**E**) The gross lung disease of mice infected with PRV-RJ. (**F**) Pathological lesions of lungs (H&E staining) from the mock- or PRV-infected mice were shown (upper). Immunohistochemistry detection of PRV-RJ-gC in mice (lower). Scale bar: 100 μm. (**G**) Histological scoring of lung injury. (**H**) Viral copies of lung tissues were detected by qPCR. (**I**) Detection of proinflammatory cytokines (TNF-α, IFN-γ, IL-6, and IL-1β) in serum by ELISA. (**J and K**) The protein levels of GSDMD-full, GSDMD-NT, pro-Caspase-1, p10, and PRV-RJ-gC in lung tissues (**J**) and in RAW264.7 cells (**K**) were analyzed by western blot. (**L**) The LDH assay in lung tissues was measured. (**M**) The LDH assay in RAW264.7 cells was measured. ***, *P* < 0.001.

Based on previous results, in order to better understand the mechanism of PRV-induced lung injury, we established a PRV-induced lung injury model in C57BL/6J mice. We intraperitoneally injected C57BL/6J mice with 2 × 10^3^ TCID_50_ PRV-RJ strain. After artificial infection by PRV-RJ for 5 days, symptoms appeared similar to natural infection. The mice began to show itching symptoms. The mice in the PRV-RJ group began to show clinical symptoms on the third day after PRV-RJ challenge, and all the mice died on the seventh day, with a survival rate of 0% (Fig. 2A). The PRV-infected WT mice suffered from gradual weight loss, and the mean body weight of the mice in the PRV-infected group dropped below 20 g at the moment of death (Fig. 2B). According to the scoring criteria of clinical symptom, the clinical score of the PRV-RJ group was 3.0 on the fourth to seventh day (Fig. 2C). In the PRV-infected lung tissues, we indeed detected a large number of PRV (Fig. 2D, F and H; Fig. S1A lower panel) and observed different degrees of lung bleeding and a typical histopathological lesion in lung tissues (Fig. 2E and F; Fig. S1A upper panel). Yet, no typical histopathological damage that could cause death in infected mice was found in other tissues (data not shown). Lung injury score was significantly increased in PRV-infected mice (Fig. 2G). These data suggested that the mice might die from lung tissue injury in the PRV-RJ group. Meanwhile, in line with the changes of PRV-infected pigs, the release levels of TNF-α, IFN-γ, IL-6, and IL-1β in lung tissues of PRV-infected mice were significantly higher than those in the mock-infected mice (Fig. 2I). Caspase-1 was similarly activated to produce active caspase-1 (p10), and GSDMD was similarly cleaved to produce the N-terminal cleavage fragments of GSDMD (GSDMD-NT) in PRV-infected lung tissues (Fig. 2J), as well as in PRV-infected RAW264.7 cells (Fig. 2K). The release level of LDH was significantly increased in PRV-infected lung tissues, as well as in PRV-infected RAW264.7 cells (Fig. 2L and M). Taken together, these results indicated that PRV infection activates inflammatory responses and causes the GSDMD-mediated pyroptosis in mice.

### GSDMD-mediated pyroptosis plays a pivotal role in PRV induction of inflammatory responses and lung tissue injury

**Fig 3:**
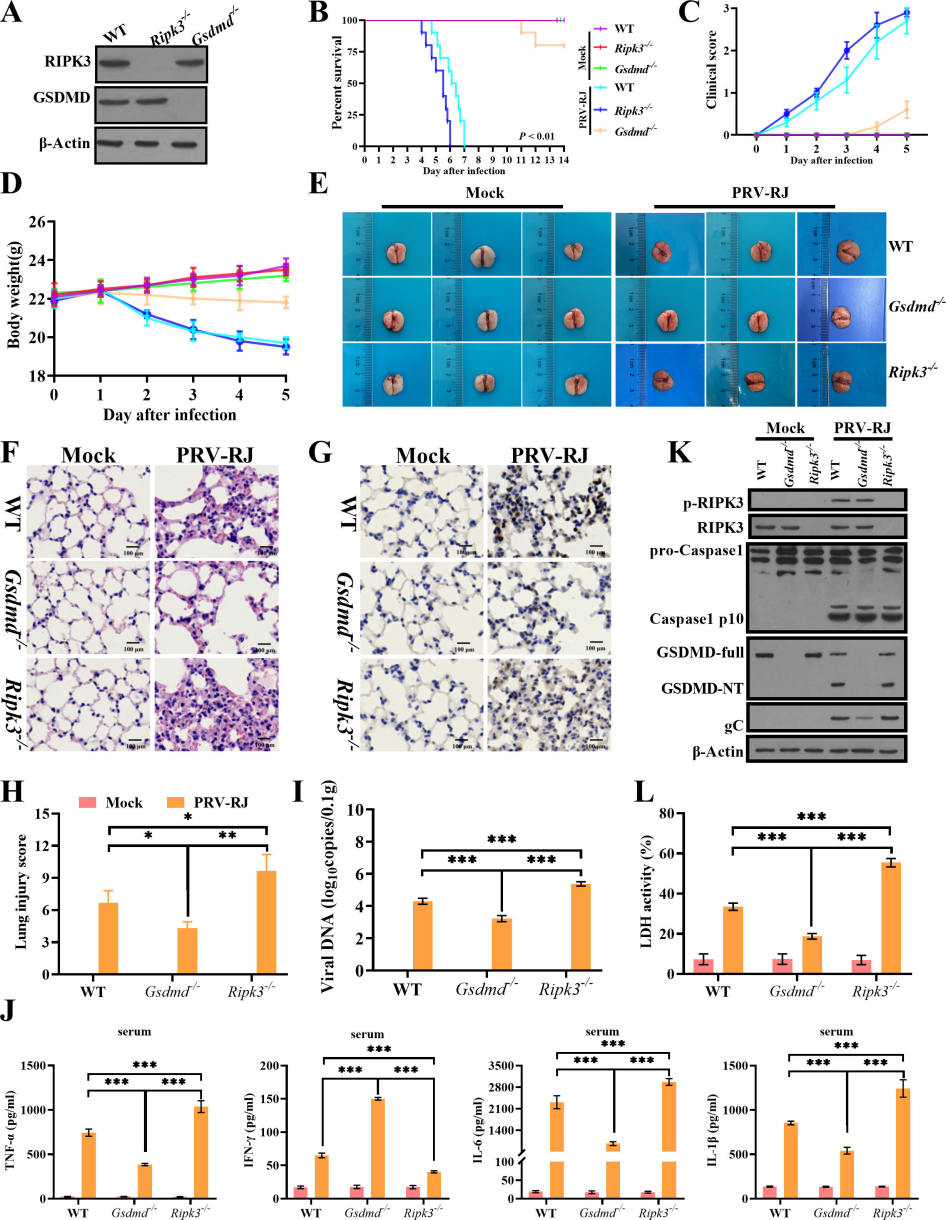
GSDMD deficiency enhances host defense against PRV infection *in vivo*.(**A**) The lung tissues of WT mice, *Ripk3*^*-/-*^ mice and *Gsdmd*^*-/-*^ mice were detected by RIPK3 and GSDMD antibody. (**B to D**) Six-week-old male WT mice, *Ripk3*^*-/-*^ mice and *Gsdmd*^*-/-*^ mice (n = 10 per group) were inoculated intraperitoneally with PBS or 2 × 10^3^ TCID_50_ PRV-RJ. Survival rate (**B**), Clinical score (**C**) and Body weight (**D**) of mice infected with PRV-RJ. (**E to L**) Six-week-old male WT mice, *Ripk3*^*-/-*^ mice and *Gsdmd*^*-/-*^ mice (n = 6 per group) were inoculated intraperitoneally with PBS or 2 × 10^3^ TCID_50_ PRV-RJ, and then all mice were sacrificed on the 3^rd^ day. (**E**) The gross lung disease of mice infected with PRV-RJ. (**F**) Pathological lesions of lungs (H&E staining) from the different mice were shown. Scale bar: 100 μm. (**G**) Immunohistochemistry detection of PRV-RJ-gC in mice. Scale bar: 100 μm. (**H**) Histological scoring of lung injury. (**I**) Viral copies of lung tissues were detected by qPCR. (**J**) Detection of proinflammation cytokines (TNF-α, IFN-γ, IL-6 and IL-1β) in serum by ELISA. (**K**) The protein level of RIPK3, p-RIPK3, pro-Caspase1, p10, GSDMD-full, GSDMD-NT and PRV-gC, β-Actin in lungs tissues were analyzed by western blotting. (**L**) The LDH assay in lung tissues were measured. *, *P* <0.05; **, *P* <0.01; *** *P* < 0.001.

There are many ways of cell death, but the most common ways in inflammation-induced cell death are necroptosis and pyroptosis. Although we observed GSDMD-mediated pyroptosis in the PRV-induced lung injury, it is not clear whether necroptosis is also involved in PRV-induced lung injury. We attempted to use the *Gsdmd^−/−^* mice and the *Ripk3^−/−^* mice to verify this hypothesis (Fig. 3A). *Gsdmd^−/−^* mice and *Ripk3^−/−^* mice are viable and fertile with no overt pathology under stress-free conditions (data not shown). The inoculation scheme remained the same as that used in the wild-type mice, and the severity of infection in mice was monitored for 14 days. PRV infection generally resulted in rapid weight loss by approximately day 2 p.i., and this was accompanied by a gradual worsening of symptoms and substantial mortality in *Ripk3^−/−^* mice, similar to WT mice. However, almost all of the PRV-infected *Gsdmd^−/−^* mice survived and exhibited mild clinical symptoms (Fig. 3B through D), suggesting that the deficiency of GSDMD reduces the severity of disease upon lethal PRV infection rather than the deficiency of RIPK3.

Meanwhile, PRV-infected *Gsdmd^−/−^* mice revealed milder pathological damage and inflammation (i.e., the accumulation of inflammatory cells, including lymphocytes) and lung injury score in the lower respiratory tract (bronchiole and alveoli) than PRV-infected *Ripk3^−/−^* mice (Fig. 3E, F and H; Fig. S2A). PRV-RJ antigen-positive tissue and the viral DNA copies in the *Gsdmd^−/−^* mice lungs (average virus DNA copies, 10^3.48^ copies/0.1 g) were lower than those in the *Ripk3^−/−^* mice lungs and WT mice lungs (Fig. 3G and I; Fig. S2B). Similarly, the levels of pro-inflammatory cytokines (TNF-α, IL-6, and IL-1β) were lowest in PRV-infected *Gsdmd^−/−^* mice compared with *Ripk3^−/−^* mice and WT mice (Fig. 3J). Conversely, the level of IFN-γ was highest in PRV-infected *Gsdmd^−/−^* mice compared with *Ripk3^−/−^* mice and WT mice (Fig. 3J). In *Ripk3^−/−^* mice and WT mice, the cleavage of GSDMD (GSDMD-NT) was able to be detected similarly after PRV infection. Cleaved caspase 1 (p10) was detected in WT mice, *Ripk3^−/−^* mice, and *Gsdmd^−/−^* mice after PRV infection, while the RIPK3 protein was also activated by PRV infection in the lung tissues of *Gsdmd^−/−^* mice as well as that in WT mice (Fig. 3K). The level of LDH activity was lowest in PRV-infected *Gsdmd^−/−^* mice compared with *Ripk3^−/−^* mice and WT mice (Fig. 3L). These data demonstrated that GSDMD plays a pivotal role in the process of PRV inducing tissue injury and causing death of infected mice and suggested that PRV infection might promote the injury of lung tissue through inducing GSDMD-mediated pyroptosis rather than RIPK3-mediated necroptosis.

### The level of NK cells is higher in *Gsdmd^−/−^* mice than in wild-type mice during PRV infection

**Fig 4:**
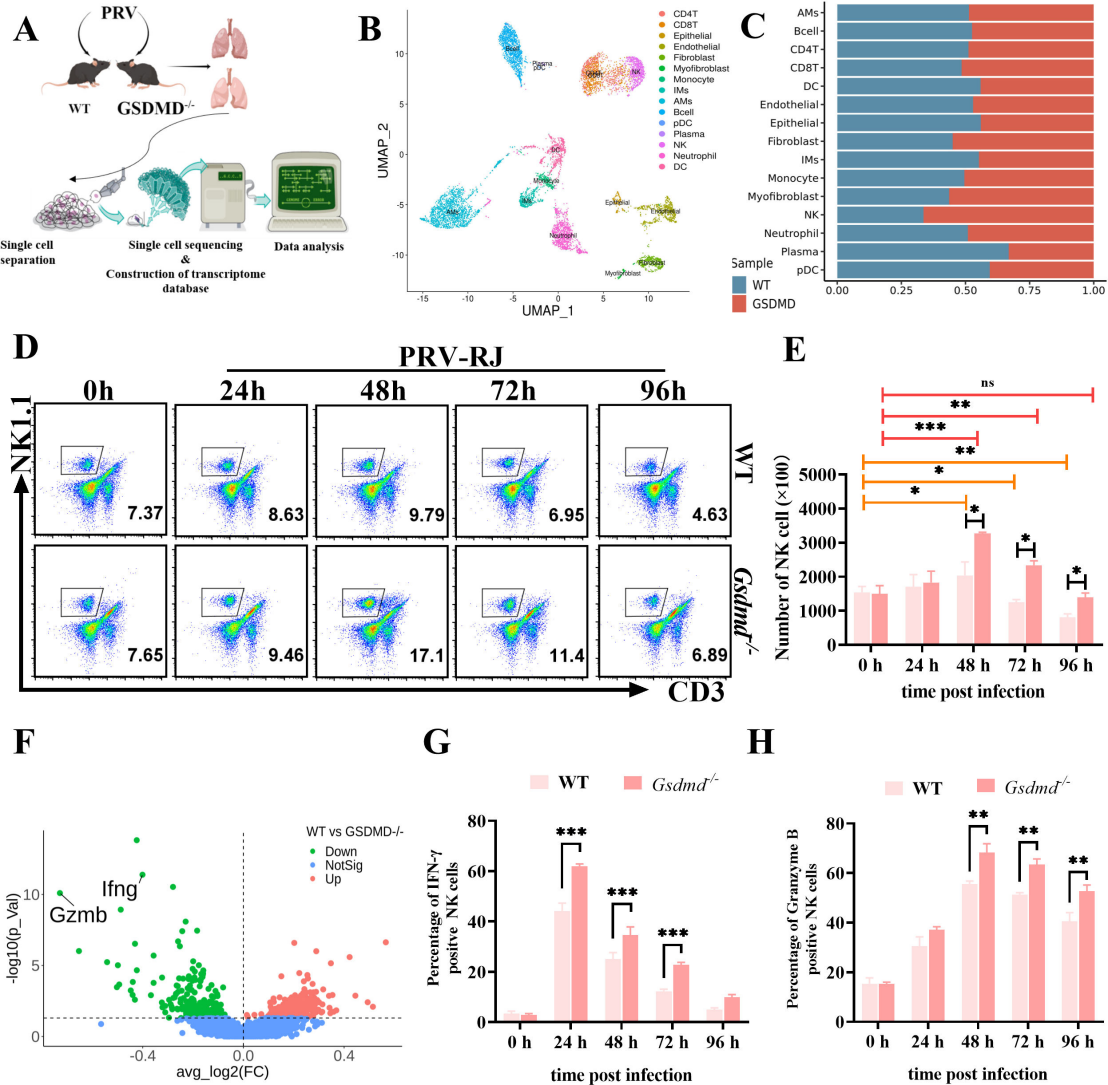
Single cell transcriptional profiling of WT mice and *Gsdmd^−/−^* mice immune cells in PRV infection caused lung injury. (**A**) Schematic of experimental workflow for defining and comparing immune transcriptional states in WT mice and *Gsdmd^−/−^* mice after PRV infection. (**B**) UMAP representation of 13,275 single cells grouped into 22 distinct cell types and the cell types identified by marker genes in both PRV-infected WT mice and PRV-infected *Gsdmd^−/−^* mice. (**C**) Proportion of immune cells among PRV-infected WT and PRV-infected *Gsdmd^−/−^* lung tissues at 48 h. (D–H) WT mice (n = 6) and *Gsdmd^−/−^* (n = 6) mice were infected with 2 × 10^3^ TCID_50_ PRV-RJ for 0, 24, 48, 72, and 96 h. (**D**) Flow cytometry was used to evaluate the NK cells (CD3^-^NK1.1^+^) in the lungs of WT mice and *Gsdmd^−/−^* mice. (**E**) Quantification of the number of NK cells. (**F**) Differential gene expression of NK cells in PRV-infected WT mice and in PRV-infected *Gsdmd^−/−^* mice was shown in a volcano plot. (G and H) The percentage of IFN-γ (**G**) and Granzyme B (**H**) positive NK cells from the lung single-cell suspension of PRV-infected WT mice and PRV-infected *Gsdmd^−/−^* mice. ns, no significant difference; *, *P* < 0.05; **, *P* < 0.01; ***, *P* < 0.001.

To further explain why GSDMD deletion can significantly weaken the pathogenic ability of PRV and explore what role GSDMD plays and how GSDMD acts in the process of PRV infection and induction of tissue injury, we first sequenced the transcriptome of over 13,275 unselected cells of inflammatory cells isolated from the lung of PRV-infected WT mice and PRV-infected *Gsdmd^−/−^* mice using scRNA-seq (Fig. 4A; Table S1). A data set of 9,872 high-quality cells was used for subsequent data analysis, and all the cells were clustered into 22 clusters through Uniform Manifold Approximation and Projection for Dimension Reduction (UMAP) (Fig. 4B; Table S2). We categorized these clusters into plasma cells, alveolar macrophage cells, NK cells, T lymphocytes, B lymphocytes, macrophages, monocytes and DC cells, neutrophils, mix-cycling cells, fibroblasts, myofibroblasts, endothelial cells, and epithelial cells using the canonical markers in each cluster (Fig. 4B; Table S2). Among these cells, only NK cells appeared to have a significant increase in the *Gsdmd^−/−^* mice relative to WT mice (Fig. 4C). In line with this finding, flow cytometry detection also showed that the percentage and number of NK cells were higher in PRV-infected *Gsdmd^−/−^* mice than in PRV-infected WT mice at 48, 72, and 96 h post-infection (Fig. 4D and E). While the percentage and number of NK cells were markedly increased in WT mice at 48 h post-PRV infection relative to 0 h, they decreased in WT mice at 72 and 96 h post-PRV infection relative to 0 h; but the percentage and number of NK cells were significantly increased in *Gsdmd^−/−^* mice at 48 and 72 h post-PRV infection relative to 0 h, and no significant difference in *Gsdmd^−/−^* mice at 96 h post-PRV infection relative to 0 h (Fig. 4D and E). Based on these results, it is suggested that PRV infection might activate GSDMD-mediated NK pyroptosis. INF-γ and granzyme B, as the typical marker molecules for NK cell functional activity, were significantly decreased in PRV-infected WT mice compared with those in PRV-infected *Gsdmd^−/−^* mice in volcanic map analysis (Fig. 4F), as well as in flow cytometry analysis (Fig. 4G and H; Fig. S3). These results suggested that PRV infection leads to a decrease in the number and functional activity of NK cells, while the absence of GSDMD can significantly alleviate the decrease in NK cell number and functional activity caused by PRV infection.

### NK cells play a crucial role in the control of PRV infection and PRV-induced lung injury

**Fig 5:**
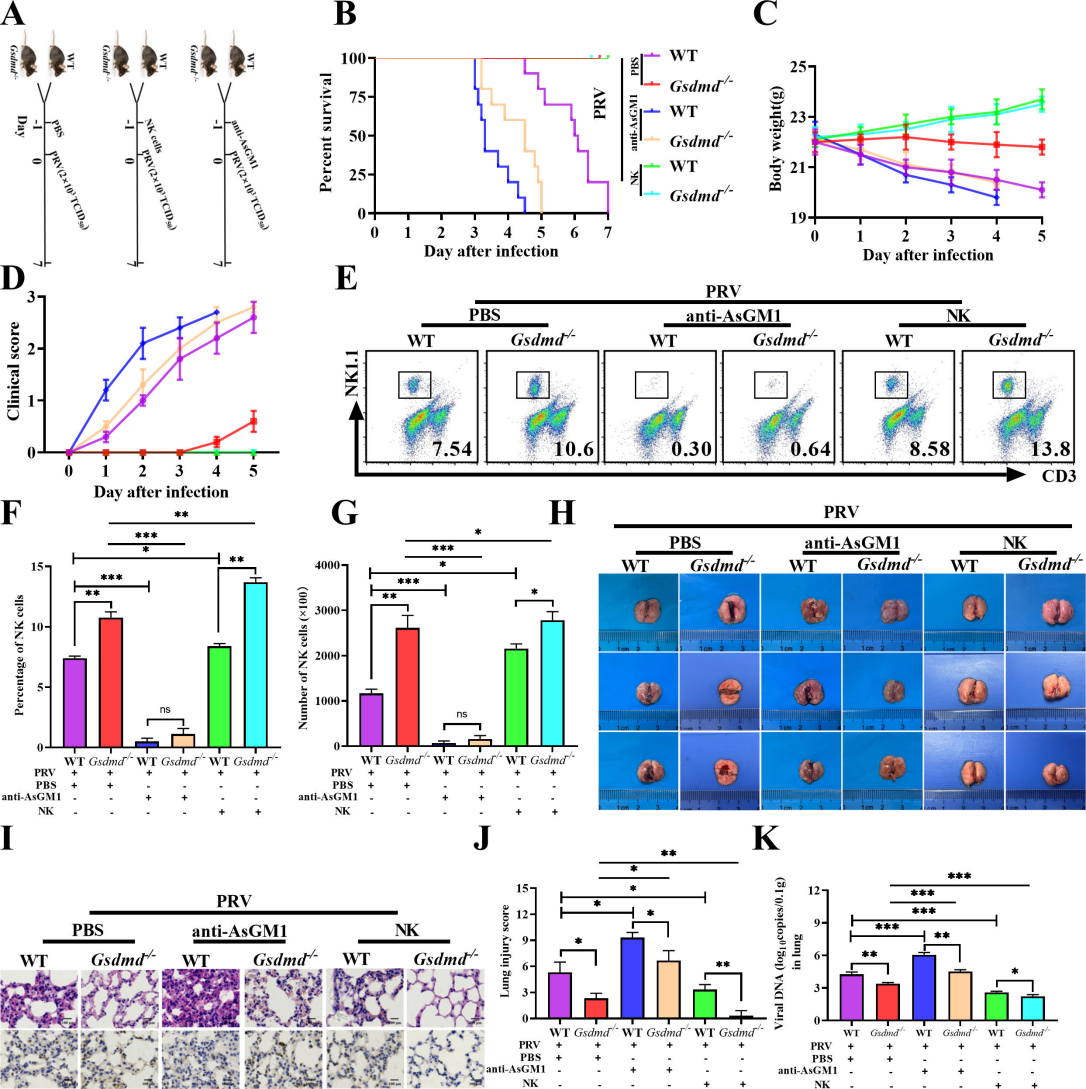
NK cells antagonize PRV infection *in vivo*. (**A**) Therapeutic preventive strategy for PRV challenge and NK treatment in mice. Mice were randomly divided into three groups. AsGM1 group: WT mice (n = 10) and *Gsdmd^−/−^* mice (n = 10) were injected with 200 μg anti-AsGM1 via tail vein; NK group: WT mice (n = 10) and *Gsdmd^−/−^* (n = 10) mice were injected with 5 × 10^5^ NK cells (0.2 mL) via tail vein; PBS group: WT mice (n = 10) and *Gsdmd^−/−^* (n = 10) mice were injected with 0.2 mL PBS via tail vein. One day later, all mice were infected with 2 × 10^3^ TCID_50_ PRV-RJ for 7 days. Survival rate (**B**), body weight (**C**), and clinical score (**D**) of the different groups of mice were analyzed. (E–K) WT mice (n = 6) and *Gsdmd^−/−^* mice (*n* = 6) were treated as in A, then all mice were sacrificed on the third day. (**E**) Flow cytometry was used to evaluate the NK cells (CD3^-^NK1.1^+^) in the lungs of WT mice and *Gsdmd^−/−^* mice. (**F**) The percentage of NK cells in each group was statistically analyzed. (**G**) The number of NK cells in each group was statistically analyzed. (**H**) Gross lesions of lung tissues in each group were as shown. (**I**) Pathological lesions of lungs (H&E staining) from the different groups of mice were shown (upper), and immunohistochemistry detection of PRV-RJ-gC in mice (lower). Scale bar: 100 μm. (**J**) Histological scoring of lung injury. (**K**) Viral copies of lung tissues were detected by qPCR. *, *P* < 0.05; **, *P* < 0.01; ***, *P* < 0.001.

To clarify the relationship between NK cells and PRV infection-induced lung injury, we observed and compared the survival rate, body weight, and lung tissue lesions of the WT mice and *Gsdmd^−/−^* mice that eliminated NK cells by injecting anti-AsGM1 (AsGM1 group), the WT mice and *Gsdmd^−/−^* mice that supplemented NK cells (NK group), and the control WT mice and *Gsdmd^−/−^* mice that were injected with the same volume of PBS (PBS group) (Fig. 5A). As we expected, after PRV infection, the death time of AsGM1 group WT mice was earlier than that of PBS group WT mice (3–5 days vs 5–7 days); the death time of AsGM1 group *Gsdmd^−/−^* mice was similar to that of PBS group WT mice (5–7 days), which was different from PBS group *Gsdmd^−/−^* mice where no death occurred (Fig. 5B); AsGM1 group WT mice also experienced earlier weight loss and appeared more severe clinical symptoms in the early phase of PRV infection (Fig. 5C and D). However, it was worth noting that no visible clinical symptoms or death were observed in both the NK group WT mice and the NK group *Gsdmd^−/−^* mice after PRV infection (Fig. 5B through D). Flow cytometry detection showed that injecting anti-AsGM1 or injecting NK cells indeed could eliminate or increase NK cell numbers in corresponding groups (Fig. 5E through G). Consistent with the clinical changes and NK number changes, eliminating NK cells by injecting anti-AsGM1 exacerbated lung tissue lesions and lung injury score caused by PRV infection in both WT mice and *Gsdmd^−/−^* mice, whereas supplementing NK cells alleviated lung tissue lesions and lung injury score caused by PRV infection in both WT mice and *Gsdmd^−/−^* mice (Fig. 5H through J; Fig. S4A in upper panel). These results indicated that NK cells play a crucial role in relieving the PRV-induced lung tissue damage.

To clear the roles of NK cells during PRV infection, we detected and compared the PRV titers in lung tissues of the AsGM1 group, the NK group, and the PBS group. As shown in Fig. 5I and K; Fig. S4A (lower panel), among all of the groups, PRV protein and DNA levels were highest in lung tissue (approximately 10^6^ copies/0.1 g of tissue) of the AsGM1 group WT mice, while PRV protein and DNA levels were lowest in lung tissue (approximately 10^2.2^ copies/0.1 g of tissue) of the NK group *Gsdmd^−/−^* mice. Whether WT mice or *Gsdmd^−/−^* mice, removing NK cells increased the PRV infection level, while recovering NK cells decreased the PRV infection level (Fig. 5I and K; Fig. S4A in lower panel). Taken together, these data suggested that either increasing or decreasing NK cells significantly affects the replication of PRV, and that NK cells execute a crucial role in either resisting PRV infection or relieving the lung tissue damage induced by PRV.

### PRV infection excessively activates macrophages to secrete more TNF-α to induce NK cell depletion to facilitate viral infection and aggravate viral pathogenicity

**Fig 6:**
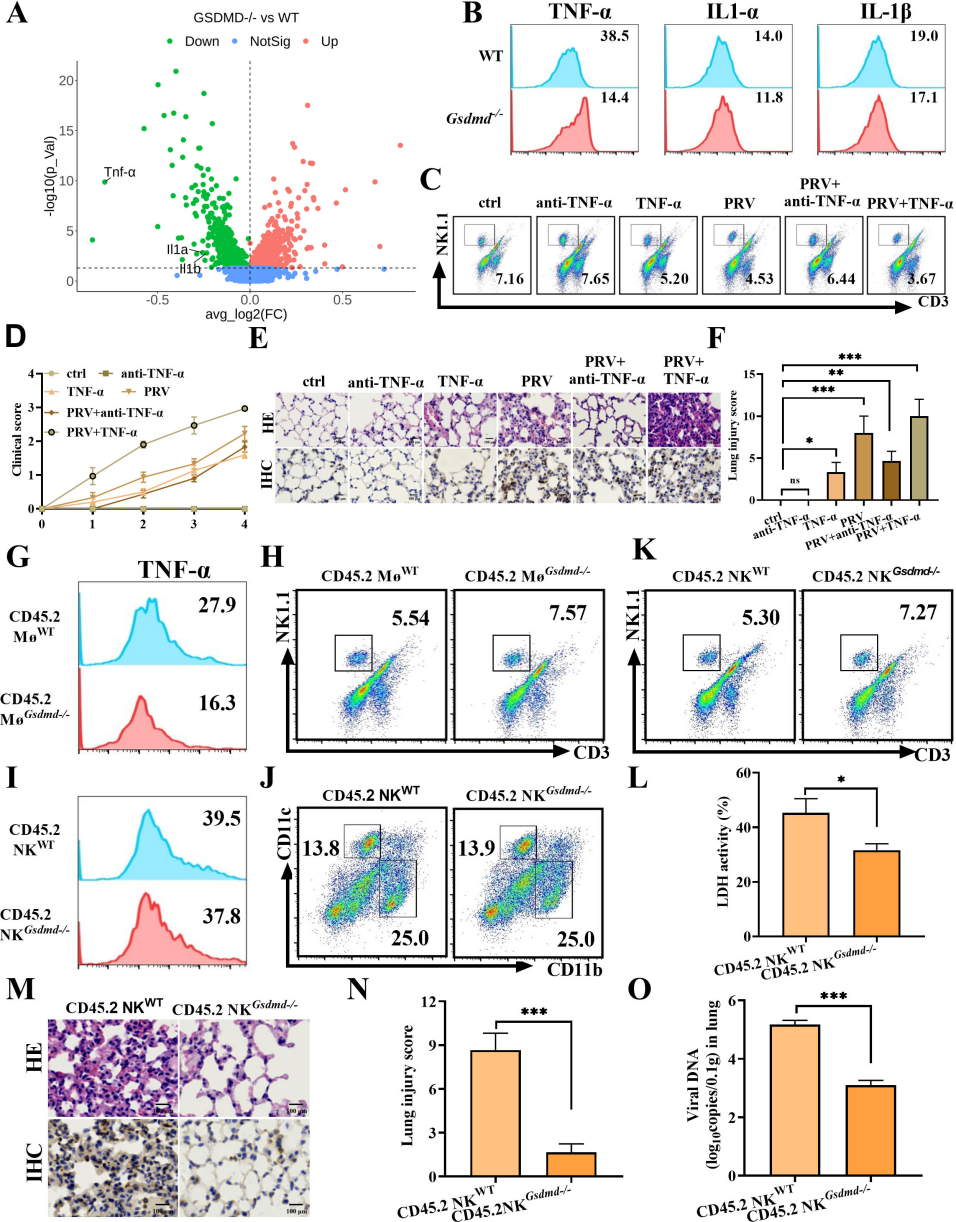
Macrophages release TNF-α to activate NK cells to exacerbate lung injury during PRV infection. (**A**) Differential gene expression in AMs of PRV-infected WT mice and PRV-infected *Gsdmd^−/−^* mice was shown in a volcano plot. (**B**) WT mice (n = 6) and *Gsdmd^−/−^* mice (n = 6) were infected with 2 × 10^3^ TCID_50_ PRV for 4 days, and the level of TNF-α, IL-1α, and IL-1β was detected by flow cytometry. (**C through F**) WT mice (n = 6) were injected with 500 μg of neutralizing antibody against TNF-α or 4 ng of TNF-α. Twelve hours later, mice were intraperitoneally injected with 2 × 10^3^ TCID_50_ PRV for 4 days. (**C**) The percentage of NK cells was detected by flow cytometry in each corresponding group. (**D**) The clinical score of each corresponding group. (**E**) Pathological lesions of lungs (H&E staining) from the corresponding group were shown (upper). Immunohistochemistry detection of PRV-RJ-gC in each corresponding group (lower). Scale bar: 100 µm. (**F**) Histological scoring of lung injury. (G and H) Mice were treated as method and Fig. S5B. The percentage of TNF-α positive macrophages (**G**) and the percentage of NK cells (**H**) were detected by flow cytometry. (**I through O**) Mice were treated as method and Fig. S5D. The percentage of TNF-α positive macrophages (**I**), the percentage of macrophages (**J**), and the percentage of NK cells (**K**) were detected by flow cytometry. (**L**) The LDH assay in supernatants was measured. (**M**) Pathological lesions of lungs (H&E staining) from the different groups of mice were shown (upper). Immunohistochemistry detection of PRV-RJ-gC in mice (lower). Scale bar: 100 µm. (**N**) Histological scoring of lung injury. (**O**) Viral copies of lung tissues were detected by qPCR. *, *P* < 0.05; **, *P* < 0.01; ***, *P* < 0.001.

The functional weakening of NK cells can weaken the body’s ability to eliminate foreign infectious agents (22), which leads to the overactivation of macrophages and the release of a large number of inflammatory cytokines, such as TNF-α and IL-1, thereby causing inflammatory tissue damage (23). To differentiate the altered gene in PRV-infected WT and *Gsdmd^−/−^* mice, we performed volcano plot analysis with a threshold of *P* < 0.05 in alveolar macrophages (AMs). Among the 8,240 genes, 74 genes (32 increased genes and 42 decreased genes) were significantly different in the AMs of PRV-infected *Gsdmd^−/−^* mice and the AMs of PRV-infected WT mice. The levels of *Il1a*, *Tnf,* and *Il1b* were significantly downward revisions in the AMs of PRV-infected *Gsdmd^−/−^* mice (Fig. 6A). In addition, KEGG analysis of Fabp4^+^ AMs showed that NF-κB and TNF-α signaling pathways were activated in PRV-infected cells (Fig. S5A). These results suggested that PRV infection promoted macrophages to M1 polarization and might result in the overactivation of macrophages. Flow cytometry detection also showed that the macrophages released larger TNF-α in PRV-infected WT mice than in PRV-infected *Gsdmd^−/−^* mice (Fig. 6B; Fig. S5B). As we expected, neutralizing antibody against TNF-α injection could rescue the reduction of NK cells caused by PRV infection, whereas injection of TNF-α also further exacerbated the reduction of NK cells caused by PRV infection (Fig. 6C). According to the scoring criteria of clinical symptoms, the clinical score of the PRV +anti-TNF-α group was approximately 2.0 on the 4th day, and the clinical score of the PRV + TNF-α group was approximately 3.0 on the fourth day (Fig. 6D). Neutralizing antibody against TNF-α injection could alleviate lung tissue lesions and lung injury score caused by PRV infection, whereas injection of TNF-α also further aggravated lung tissue lesions and lung injury score caused by PRV infection (Fig. 6E and F). Taken together, those data suggested that PRV infection caused macrophages to secrete large amounts of TNF-α, which led to a decrease in the number of NK cells and consequently resulted in lung injury.

To further confirm whether macrophages affect the number and function of NK cells during PRV infection, a total of 1 × 10^5^ macrophage cells sorted from either WT mice or *Gsdmd^−/−^* mice (both the background of CD45.2) were adoptively transferred to clodronate liposomes-treated WT mice (the background of CD45.1), respectively. After 24 h, the two groups of mice were infected with PRV, and the NK cells were detected by flow cytometry 96 h later (Fig. S5C). Clodronate liposomes, an inhibitor of macrophages, were able to clear macrophages from mice (Fig. S5D). The percentage of TNF-α positive macrophages (16.3%) in the mice transferred *Gsdmd^−/−^* macrophages was significantly lower than that (27.9%) in the mice transferred WT macrophages, while the percentage of NK cells (7.57%) in the mice transferred *Gsdmd^−/−^* macrophages was significantly higher than that (5.54%) in the mice transferred WT macrophages (Fig. 6G and H). In addition, a total of 1 × 10^5^ NK cells sorted from either WT mice or *Gsdmd^−/−^* mice (both the background of CD45.2) were adoptively transferred to anti-AsGM1-treated WT mice (the background of CD45.1), respectively. After 24 h, the two groups of mice were infected with PRV, and the macrophage cells were detected by flow cytometry 96 h later (Fig. S5E). Anti-AsGM1, an inhibitor of NK cells, was able to clear NK cells from mice (Fig. S5F). However, the percentage of TNF-α positive macrophages and the percentage of macrophage cells did not show a significantly different result between the mice transferred with *Gsdmd^−/−^* NK cells and the mice transferred with WT NK cells (Fig. 6I and J). Yet, the percentage of NK cells in the mice transferred *Gsdmd^−/−^* NK cells was higher than that in the mice transferred WT NK cells (Fig. 6K). As a result, the LDH yield of lung tissue in the mice transferred *Gsdmd^−/−^* NK cells was lower than that in the mice transferred WT NK cells. The data demonstrated that PRV infection induced GSDMD-mediated NK cell pyroptosis (Fig. 6L); the lung tissue lesions caused by PRV infection and the positive signal of PRV gC and lung injury score were markedly improved in the mice transferred *Gsdmd^−/−^* NK cells compared with the mice transferred WT NK cells (Fig. 6M and N); PRV DNA levels in lung tissue were also markedly reduced in the mice transferred *Gsdmd^−/−^* NK cells compared with the mice transferred WT NK cells (Fig. 6O). Taken together, these data demonstrated that the overactivation of macrophages can induce the decrease of NK cells via TNF-α in PRV-infected WT mice, and that GSDMD deletion can inhibit the overactivation of macrophages, reduce the release of TNF-α, and prevent the reduction of NK cell number.

### NSA relieves the lung injury in WT mice caused by PRV infection

**Fig 7:**
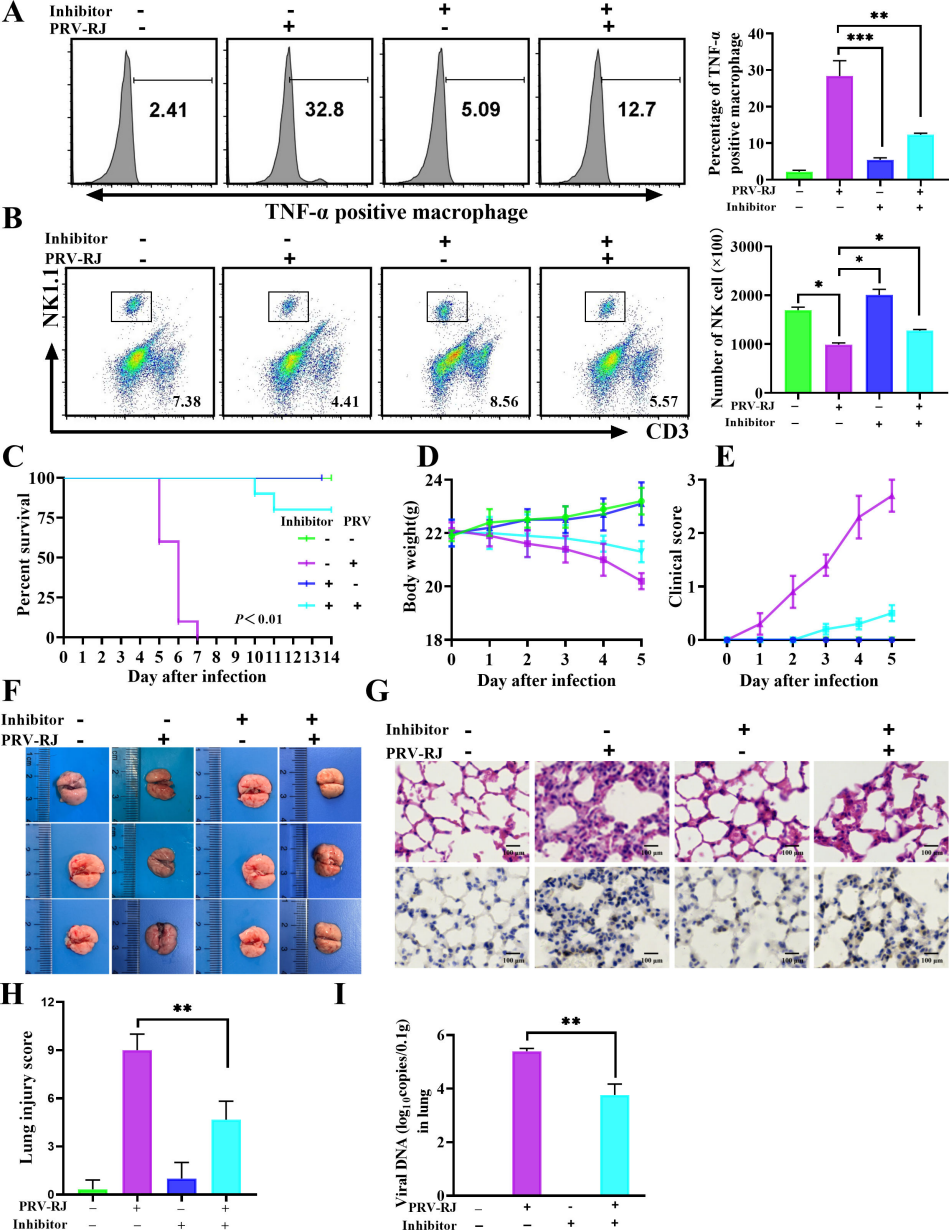
Inhibition of GSDMD can effectually alleviate PRV pathogenicity *in vivo*. (**A and B**) Six-week-old WT mice were randomly divided into four groups, six mice per group. WT mice were injected with 100 μg necrosulfonamide (NSA, inhibitor of GSDMD) via the abdominal cavity, followed by 2 × 10^3^ TCID_50_ PRV infection for 4 days at 2 h post-injection. The percentage of TNF-α positive macrophages (**A**) and the percentage and number of NK cells (**B**) were detected by flow cytometry. (C–E) Mice were treated as in A, 10 mice per group. Survival rate (**C**), body weight (**D**), and clinical score (**E**) were monitored. (F–I) Mice were treated as in A, and then all mice were sacrificed on the fourth day, six mice per group. (**F**) Gross lesions of the lungs in each group. (**G**) Pathological lesions of the lung (H&E staining) from the different groups of mice were shown (upper panel), and immunohistochemistry detection of PRV-RJ-gC in mice (lower panel). Scale bar: 100 μm. (**H**) Histological scoring of lung injury. (**I**) Viral copies of lung tissues were detected by qPCR. *, *P* < 0.05; **, *P* < 0.01; ***, *P* < 0.001.

Based on the above deduction, we observed whether the inhibitor of GSDMD, NSA, could reduce the release of TNF-α and prevent the reduction of NK cell depletion to enhance the body’s ability to resist infections and to alleviate the lung tissue damage induced by PRV infection. WT mice were injected with 100 µg of NSA via enterocoelia, followed by PRV infection at 2 h post-injection according to previous studies (24). At 96 h post-infection, the percentage of TNF-α-positive macrophages (12.7%) in PRV-infected mice with NSA treatment was markedly lower than that in PRV-infected mice without NSA treatment (32.8%) (Fig. 7A). Meanwhile, the levels of TNF-α in lung tissue and blood were also significantly decreased in PRV-infected mice with NSA treatment relative to PRV-infected mice without NSA treatment (Fig. S6A and B). Consistently, the percentage and number of NK cells were markedly increased in PRV-infected mice with NSA treatment relative to PRV-infected mice without NSA treatment (Fig. 7B). In the observation period of 14 days, the mice without NSA treatment began to show clinical symptoms on the third day after PRV challenge, and all the mice died on the seventh day, with a survival rate of 0%, whereas only part of the NSA-treated mice began to die on the ninth day after PRV challenge, with a survival rate of 80% (Fig. 7C). Meanwhile, the NSA-treated mice only exhibited a mild weight loss compared to the mice without NSA treatment on the third day after PRV challenge and exhibited a slower weight loss compared to the mice without NSA treatment (Fig. 7D); the NSA-treated mice only exhibited milder clinical change compared to the mice without NSA treatment on the third to fifth day after PRV challenge (Fig. 7E). Consistent with the clinical changes, NSA treatment significantly alleviated lung tissue lesions and lung injury score caused by PRV infection (Fig. 7F through H; Fig. S6C), and reduced the viral infection (Fig. 7G and I; Fig. S6C). The virus DNA copies in the PRV-infected lungs without NSA were 10^5.76^ copies/0.1 g (average virus DNA copies), whereas the average virus DNA copies were 10^3.76^ copies/0.1 g in the PRV-infected lungs with NSA treatment (Fig. 7I). Taken together, these data demonstrated that inhibition of GSDMD-mediated NK cell depletion can effectually alleviate PRV-induced pathogenicity *in vivo*, and NSA is expected to be a candidate agent for the prevention and control of PRV infection.

## DISCUSSION

Since pigs and a variety of animals are often associated with lung inflammation after PRV infection, causing lung damage, presenting with mild to severe interstitial pneumonitis and hemorrhagic pneumonitis (25). Therefore, the lung may be the main target organ of PRV infection, and it is of great significance to investigate the mechanisms of PRV infection-inducing the inflammatory injury of lung tissue and the roles of inflammatory cells and cytokines in this process. In our study, we found that GSDMD-mediated NK cell pyroptosis played a crucial role in PRV infection-induced interstitial pneumonia. First of all, we confirmed that PRV infection could cause pyroptosis of lung tissue in piglets. Conversely, deficiency of GSDMD alleviated the lung injury and increased the survival rate of PRV-infected mice rather than deficiency of RIPK3. However, the regulatory role of GSDMD in the formation of interstitial pneumonia caused by PRV infection is unclear. Furthermore, single-cell sequencing analysis of the difference between GSDMD-deficient mice and WT mice showed that NK cells might play an important role in the process of fighting viral infection. PRV infection induced the overactivation of macrophages to secrete TNF, to overactivate the NK cells, resulting in the impairment of NK number and function. Hence, our finding demonstrates a novel distinct mechanism of PRV infection causing NK cell depletion following interstitial pneumonia and highlights the importance of GSDMD in PRV infection induction of lung tissue damage.

Lung injury in pigs is a pathological condition characterized by damage to the pulmonary tissue, leading to abnormal lung structure and function (26). This condition can arise from a variety of etiological factors, including viral infections, bacterial infections, parasitic infestations, environmental stressors, and immune responses (26). Inflammation is a frequent and pivotal pathological hallmark of lung injury, typified by the thickening of alveolar walls and the infiltration of inflammatory cells, including neutrophils and macrophages. Moreover, the release of inflammatory mediators, such as interleukin and tumor necrosis factor, can exacerbate the lung injury and perpetuate the inflammatory cascade (27). In our study, we found that PRV infection caused lung injury, which was characterized by significant alveolar wall thickening, infiltration of inflammatory cells, and the release of key inflammatory cytokines such as TNF-α and IL-1β. Lung injury in pigs represents a multifaceted pathological condition, precipitated by a diverse array of factors, that exerts a profound influence on the health and production performance of pigs. Nevertheless, the precise mechanisms underlying PRV-induced lung injury remain to be elucidated and warrant further investigation.

The body maintains a dynamic balance between cell proliferation and cell death, which plays a significant role in the physiopathological processes of multicellular organisms (28). Cell death is usually categorized as non-programmed cell death and programmed cell death (PCD) (29). Pyroptosis is a type of inflammation PCD (8). In recent years, GSDMD has been widely studied as a key executive molecule of pyroptosis (30). In the antibacterial and antiviral processes, bacteria and inflammasomes can cause cleavage of GSDMD by activating caspases, thereby inducing pyroptosis, which can eliminate pathogens and prevent infection. But when they are overactivated, they will trigger a cascade of inflammatory responses and cause damage to the body (31). Studies have shown that pyroptosis is closely related to the occurrence and development of a variety of infectious diseases and neurocancers, and if it is suppressed, it can effectively alleviate the exacerbation of disease-modifying diseases (32). These studies suggest that GSDMD contributes to the pathogenicity of viruses. Here, we show that PRV infection induced an increase in GSDMD-NT. And the survival rate of mice in *Gsdmd^−/−^* mice was higher than WT mice. Compared with WT mice after PRV infection, the extent of lung tissue damage was significantly relieved in *Gsdmd^−/−^* mice infected with PRV.

Necroptosis promotes further cell death and neuroinflammation in the pathogenesis of several neurodegenerative diseases (33, 34). It is mediated by RIPK1, RIPK3, and mixed lineage kinase domain-like protein (9, 35). Necroptosis has been reported to cause inflammation and severe tissue necrosis (36). However, whether necroptosis acts as a crucial mechanism during PRV infection and induces tissue damage is not well understood. Although mice lacking these kinases have very different phenotypes. RIPK1-deficient mice die soon after birth, whereas RIPK3-deficient mice are healthy (37). In our study, we used the *Ripk3^−/−^* mice to verify this hypothesis. We found that the survival rate, weight loss, and clinical score of mice didn’t improve in *Ripk3^−/−^* mice infected with PRV compared to the WT mice infected with PRV. Although PRV is closely related to HSV-1, the role of necroptosis in viral pathology might be different in its natural host (38).

GSDMD is a protein that plays a crucial role in the process of cellular pyroptosis. GSDMD is expressed in a variety of cells, including gastrointestinal epithelium, immune cells (especially macrophages and dendritic cells) (39, 40), and plays an important role in the host defense against pathogen infection and inflammatory responses. Studies have shown that GSDMD is associated with the activation and number of immune cells in inflammatory processes. For example, congenital immune cell pyrogen can exacerbate liver ischemia reperfusion injury, while GSDMD knockout can reduce the expression and production of factors in liver macrophages (41). In lung cancer, GSDMD contributes to the killing effect of cytotoxic T lymphocytes on cancer (42). GSDMD can also mediate the scorch death of pulmonary macrophages caused by *Streptococcus pneumoniae* infection. When the scorch death of pulmonary macrophages caused by infection is inhibited, it can effectively reduce the death of macrophages and alleviate the tissue injury of pneumonia (43). In addition, recent studies have shown that severe acute respiratory syndrome coronavirus 2 can induce inflammasome-dependent pyroptosis in lung epithelial cells by targeting autophagy (44). However, the role of GSDMD in interstitial pneumonia induced by PRV infection has not been reported. In this study, we provided the first evidence that the NK cells were involved in PRV-induced cell death *in vivo*. Single-cell sequencing results show that the number of NK cells in PRV-infected WT mice was lower than PRV-infected *Gsdmd^−/−^* mice. And compared with PRV-infected *Gsdmd^−/−^* mice, the function of NK cells was impaired in PRV-infected WT mice. Subsequently, we validated the single-cell sequencing results by flow cytometry, and the results were consistent. And the anti-AsGM1 (the inhibitor of NK cells) exacerbated the lung tissue damage of mice and increased the replication level of PRV. These data indicated that NK cells are the main target in PRV-induced pyroptosis, suggesting that NK cells also play important roles in modulating the anti-viral response and inflammatory balance.

NK cells are important immune effectors for preventing microbial invasion and dissemination, through natural cytotoxicity and cytokine secretion (17, 45). Immune cells are either in direct contact with each other or secrete a variety of effector substances to restrain and influence each other to maintain the normal progress of the immune response (46, 47). Macrophages of different phenotypes do act differently on NK cells (48). Cytokines such as TNF-α and IL-12 produced by M1-type macrophages promote NK cell activation (49). M2 macrophages, on the other hand, inhibit the activity and function of NK cells through IL-10 (50). In our study, according to the volcano map, the expression of *Tnf*, *Il1a*, and *Il1b* in macrophages was decreased in *Gsdmd^−/−^* mice infected with PRV rather than in WT mice infected with PRV. Next, the number of NK cells in mice treated with anti-TNF-α recovered compared to the untreated mice infected with PRV. Consistently, when PRV-infected WT mice were supplemented with macrophages from *Gsdmd^−/−^* mice, the number of NK cells was recovered. TNF abundance in the synovial fluid and tissues of patients with rheumatoid arthritis (RA) can induce pyroptosis (51), which is vital in sustained synovial inflammation by inducing nuclear factor (NF)-κB activation (52). Ling et al. (53) observed that Jinwujiangu capsules (a traditional Chinese medicine formula) can regulate NLRP3/CASPASE/GSDMD in treating RA-FLSs through pyroptosis (52). Besides, the studies have shown that IL-37 alleviates TNF-induced pyroptosis of rheumatoid arthritis fibroblast-like synoviocytes by inhibiting the NF-κB/GSDMD signaling pathway (54). Concerning the mechanism, we found that the NF-κB signaling pathway was downregulated in *Gsdmd^−/−^* mice infected with PRV using the Kyoto Encyclopedia of Genes and Genomes pathway (data not shown). Based on the above existing research, we speculated that TNF secreted by macrophages activated the NF-κB/GSDMD signaling pathway in NK cells to cause the NK cell pyroptosis in PRV-infected mice. This speculation needs to be further verified. Taken together, we considered that PRV infection leads to the overactivation of macrophages and the secretion of a large number of cytokines, such as TNF-α, which activate the NF-κB/GSDMD signaling pathway, causing pyroptosis of NK cells, impairing the ability of NK cells to resist viruses.

In conclusion, our data provide the first evidence of the mechanism by which PRV infection causes lung injury. Meanwhile, we found that pyroptosis of NK cells played an important role in limiting PRV infection. And we demonstrated that the over-activated macrophage-released TNF-α to overactive NK cells caused the depletion of NK cells. However, inhibition of GSDMD-mediated NK cell depletion can effectively alleviate PRV pathogenicity *in vivo*. Considering that pyroptosis is a type of programmed cell death related to cellular leakage and inflammation, we believe that our results provide a new perspective for understanding interstitial pneumonia caused by PRV-RJ infection. Further study will focus on which subset of macrophages is the main cell population in PRV-induced NK cell depletion.

## MATERIALS AND METHODS

### Cells and viruses

African green monkey kidney cell (Vero) was maintained in our laboratory. These cells were cultured in Dulbecco’s modified Eagle’s medium (DMEM) (12100-046; Invitrogen Carlsbad, CA, USA) supplemented with 10% heat-inactivated fetal bovine serum (13011-8611; Tianhang Biotechnology, Hangzhou, China) and penicillin/streptomycin (100 U/mL) and were maintained at 37°C in a humidified atmosphere with 5% CO_2_. 3D4/21 cells (porcine alveolar macrophages, ATCC, CRI.2843) and RAW264.7 cells (mouse alveolar macrophages, ATCC, TIB-71) were maintained in RPMI 1640 with 10% FBS and cultured in a fully humidified atmosphere containing 5% CO_2_ at 37°C. PRV-RJ strain (genotype II; GenBank accession number MH582511.1) was stocked in our laboratory and propagated in Vero cells (20).

### Antibodies and reagents

The antibodies anti-β-actin (A00702) were from Genscript; anti-Caspase1 antibody (ab179515) was purchased from Abcam; anti-GSDMD (TA4012) was purchased from Abmart; Horseradish peroxidase-conjugated anti-mouse IgG (31430) and anti-rabbit IgG (31460) were provided by Invitrogen; neutralizing antibody against TNF-α (T-703) was purchased from Leinco Technologies, Inc.; TNF-α (HY-P7090) was purchased from MedChemExpress (*MCE*); Rabbit polyclonal anti-AsGM1 antibody was purchased from Wako Chemicals, USA; Necrosulfonamide (NSA, 432531-71-0) was purchased from Merck Millipore; Anti-gC of PRV-RJ polyclonal antibody was produced in our lab. Porcine IL-1β enzyme-linked immunosorbent assay (ELISA) KIT (SEKP-0001), Mouse IL-1β ELISA KIT (Mouse IL-1β ELISA KIT), Porcine IFN-γ ELISA KIT (SEKP-0010), Mouse IFN-γ ELISA KIT (SEKM-0031), Porcine IL-6 ELISA KIT (SEKP-0004), Mouse IL-6 ELISA KIT (SEKM-0007), Porcine TNF-α ELISA KIT (SEKP-0009), and Mouse TNF-α ELISA KIT (SEKM-0034) were obtained from Solarbio. LDH Activity Assay Kit (BC0685) was purchased from Solarbio.

The flow cytometry buffer, APC anti-mouse CD45 (103112, 30-F11), FITC anti-mouse CD3 (100204, 17A2), PE anti-mouse NK1.1 (156503), APC anti-mouse CD11b (101211), PE/Cy7 anti-mouse CD11c (117317), APC anti-mouse IFN-γ (502511), PE anti-mouse TNF-α (506305), APC anti-mouse Granzyme B (372203) monoclonal antibodies were purchased from BioLegend.

### Animal experiment

Six-day-old cross-bred piglets were purchased from a native herd free of PCV2, PRV, porcine parvovirus, and other major swine pathogens as determined by PCR. All piglets were housed under the same conditions and treated similarly. For the experiment presented in Fig. 1, piglets were randomly divided into two groups (n = 6 per group), and inoculated intranasally with PRV-RJ strain (4 × 10^5^ TCID_50_) or Mock (same volume of PBS), respectively. Four days post-infection, all pigs were sacrificed and necropsied. Then, the lung tissues were collected and used for follow-up experiments.

Six-week-old healthy C57BL/6J male mice were purchased from Beijing Vitonglihua Experimental Animal Technology Co., LTD. The *Gsdmd^−/−^* mice (strain: #032663) and *Ripk3^−/−^* mice (strain: 025738) were purchased from Jackson Laboratory. All mice were housed under the same conditions and treated in a similar way. Mice were infected with the PRV-RJ strain (2 × 10^3^ TCID_50_), whereas mock group mice were infected with the same volume of DMEM/PBS alone via the abdominal cavity, respectively. The lungs were prepared for further analysis. For PRV genome copies, glycoprotein B (gB)-specific absolute quantification PCR was performed. Primer sequences were as follows: *gB*-probe-F: 5′-ACGGCACGGGC GTGATC-3′; *gB*-probe-R: 5′- ACTCGCGGTCCTCGAGCA-3′; *gB*-TaqMan-probe: FAM-CTCGCGCGACCTCATCGAGCCCTGCAC-MGB.

### Western blotting analysis

Lung tissues were lysed with RIPA buffer (Beyotime, Shanghai, China). The treated cells were gathered for lysis to obtain western blotting loading samples for gel electrophoresis. Lysates were mixed with 5× protein buffer (Solarbio, Beijing, China) and boiled for 10 min. Proteins were subjected to SDS-PAGE before being transferred to a polyvinylidene difluoride membrane. After blocking in 5% nonfat milk, the membrane was incubated overnight at 4°C in the presence of specific primary antibodies. The next day, the membrane was incubated with the indicated secondary antibodies. Following this, Enhanced Chemiluminescence Substrate (Bio-Rad, Hercules, CA, USA) was utilized to develop color for observation of the protein expression.

### Enzyme-linked immunosorbent assay

The concentration of these inflammatory cytokines, including TNF-α, IFN-γ, IL-6, and IL-1β, in the lung tissue and the serum or BALF was measured using the ELISA kits according to the manufacturer’s instructions.

### Histopathological examination and lung injury score

The tissues were dissected, collected, and fixed in 4% paraformaldehyde for at least 72 h. The fixed tissues were embedded in paraffin wax and cut into 4 µm sections. The tissue sections were subjected to histopathological analysis by staining with hematoxylin and eosin according to the manufacturer’s instructions. Histopathological changes were observed under a microscope (Nikon, Japan).

We performed the Smith lung injury scoring system. The score usually includes five aspects: alveolar congestion, bleeding, inflammatory cell infiltration, alveolar septal thickening, and hyaline membrane formation. Each score ranges from 0 to 4 points. The higher the total score, the more serious the lung injury.

### Immunohistochemistry

Immunostaining was performed according to the streptavidin-biotin-peroxidase complex immunoprecipitation kit. In brief, the sections were deparaffinized and rehydrated using xylene and graded concentrations of alcohol. Endogenous peroxidase activity was inhibited with 3% hydrogen peroxide for 15 min at room temperature. Then, the antigen epitope was subsequently unmasked using a citrate buffer via incubation in a 98°C water bath for 15 min. Non-specific antigens were blocked with 5% bovine serum albumin. The sections were incubated with anti-Pseudorabies Virus antibody (No. ab3534; Abcam, England) at 4°C overnight. After washing, the tissue slides were incubated with biotinylated goat anti-rabbit IgG (No. ab205718; Abcam, England) at RT for 1 h, then stained with DAB and counterstained with hematoxylin. Finally, the slides were evaluated under a microscope (Nikon, Japan).

### Lactate dehydrogenase assay

LDH assay was performed to determine cell viability. Add 1 mL of extraction solution to 0.1 g of lung tissue or 5 million cells for ice bath homogenization, centrifuge to obtain supernatant. Then, the LDH assay was performed using LDH assay kits according to the manufacturer’s instructions.

### Sample processing and preparation for the lung single-cell suspension

WT mice and *Gsdmd^−/−^* mice (n = 4, per group) were inoculated intraperitoneally with PRV-RJ strain (2 × 10^3^ TCID_50_) for 48 h, respectively. For the lung single-cell suspension, the lungs were resuspended with PBS containing 0.1% Collagenase type I (SCR103, Sigma-Aldrich), 0.05% DNase I (D5025, Sigma-Aldrich), and 5% FBS (13011-8611, Tianhang Biotechnology). After incubation at 37°C for 60 min, the samples were pestled and filtered with a 200-mesh sieve, pelleted (300 g, 5 min), and resuspended in ACK red blood cell lysis buffer (Gibco A1049201) for 3 min, after which the buffer was inactivated by adding excess processing buffer. Cells were then filtered through a 70 µm strainer, pelleted again (300 g, 5 min), and resuspended in PBS buffer, counted, and immediately processed for single-cell RNA sequencing.

### Flow cytometry

The cells were harvested and washed with PBS, then the surface proteins of the cells were stained according to the manufacturer’s instructions. Briefly, the cells were incubated with conjugated antibodies in the dark for 30 min to stain the surface proteins and then fixed. If intracellular molecules also need to be examined, 200 µL of cell fixative is added after the supernatant is removed by cell centrifugation and set at room temperature for minutes. After washing with PBS buffer once, add 200 µL of permeable solution for 10 min. And then washing with PBS buffer once, intracellular labeled antibodies were added and left for 30 min at 4°C away from light. After the wash process, the stained cells were analyzed using BD Accuri C6 Flow Cytometry and NovoCyte 2060R Flow Cytometry.

### Adoptive transfer

Six-week-old male mice (the background of CD45.1) were injected with clodronate liposomes for 48 h via the caudal vein, and the lung tissues of WT mice and *Gsdmd^−/−^* mice (both the background of CD45.2) were harvested to prepare the single-cell suspension. WT macrophages and *Gsdmd^−/−^* macrophages (both the background of CD45.2, CD45.2 Mø^WT^, and CD45.2 Mø*^Gsdmd−/−^*) were separated from the lung single-cell suspension by flow cytometry. Adoptive macrophages (CD45.2 Mø^WT^ and CD45.2 Mø*^Gsdmd−/−^*) were given to mice (the background of CD45.1) through the caudal vein. After 24 h, mice were infected with 2 × 10^3^ TCID_50_ PRV for 96 h and then harvested lung tissues to prepare a single-cell suspension. The macrophages and NK cells were assessed by flow cytometry.

Six-week-old male mice (the background of CD45.1) were injected with anti-AsGM1 for 48 h via caudal vein, and the lung tissues of WT mice and *Gsdmd^−/−^* mice (both the background of CD45.2) were harvested to prepare the single-cell suspension. The WT NK cells and *Gsdmd^−/−^* NK cells (both the background of CD45.2, CD45.2 NK^WT^, and CD45.2 NK*^Gsdmd−/−^*) were separated from the lung single-cell suspension by flow cytometry. Adoptive NK cells (CD45.2 Mø^WT^ and CD45.2 Mø*^Gsdmd−/−^*) were given to mice (the background of CD45.1) through the caudal vein. After 24 h, mice were infected with 2 × 10^3^ TCID_50_ PRV for 96 h and then harvested lung tissues to prepare a single-cell suspension. The macrophages and NK cells were assessed by flow cytometry.

### Statistical analysis

The calculated results were presented as mean ± standard deviation. Statistical analyses were performed using two-way analysis of variance or Student’s *t*-test. The survival of animals was compared using the log-rank test. The statistical significances were defined as *P* < 0.05 (*), and the higher significance was denoted by *P* < 0.01 (**) and *P* < 0.001 (***); ns, no significant difference. GraphPad Prism 8.0 software was used to analyze the statistics in this study.

## Data Availability

Raw single-cell sequencing data generated from the PRV-infected WT mice and PRV-infected *Gsdmd*^*−/−*^ mice in this study have been submitted to the NCBI Gene Expression Omnibus (GEO; http://www.ncbi.nlm.nih.gov/geo/) under accession number GSE282594.

## SUPPLEMENTAL MATERIAL

Supplemental figure legends jvi.00415-25-s0001.docx Legends for Fig. S1 to S6.

Fig. S1 jvi.00415-25-s0002.tif Results of HE and IHC of mock-infected lung tissue and PRV-RJ-infected lung tissue.

Fig. S2 jvi.00415-25-s0003.tif Results of HE and IHC of mock-infected lung tissue and PRV-RJ-infected lung tissue from WT mice, Gsdmd^-/-^ mice, and Ripk3^-/-^ mice.

Fig. S3 jvi.00415-25-s0004.tif Levels of IFN-γ and granzyme B in NK cells after PRV infection.

Fig. S4 jvi.00415-25-s0005.tif Results of HE and IHC of lung tissues from WT mice and Gsdmd^-/-^ mice infected with PRV in PBS group, anti-AsGM1 group, and NK group.

Fig. S5 jvi.00415-25-s0006.tif Flow diagram of adoptive transfer.

Fig. S6 jvi.00415-25-s0007.tif GSDMD inhibitor can reduce the production of TNF-α induced by PRV infection.

Supplemental results jvi.00415-25-s0008.docx Three parallel results of caspase 1 from primary figures.

Supplemental tables jvi.00415-25-s0009.docx Single cell sequencing data.

Supplemental table legends jvi.00415-25-s0010.docx Legends for Tables S1 and S2.

ASM does not own the copyrights to Supplemental Material that may be linked to, or accessed through, an article. The authors have granted ASM a non-exclusive, world-wide license to publish the Supplemental Material files. Please contact the corresponding author directly for reuse.
