# Supplementary material for: Pseudorabies virus induces natural killer cell depletion by GSDMD-mediated inflammation and pyroptosis to promote infection and lung injury
Source: J Virol. 2025 Jul 24;99(8):e00415-25. doi: 10.1128/jvi.00415-25 (PMC12363163; doi:10.1128/jvi.00415-25)
Supplement: Supplemental figure legends — Legends for Fig. S1 to S6. [file jvi.00415-25-s0001.docx]

# **Fig. S1 The results of HE and IHC of mock-infected lung tissue and PRV-RJ-infected lung tissue.**

**(A)** HE and IHC results from the other two parallel experiments in Figure 2F. Scale bar: 100 μm.

# **Fig. S2 The results of HE and IHC of mock-infected lung tissue and PRV-RJ-infected lung tissue from WT mice, *Gsdmd^-/-^* mice and *Ripk3^-/-^* mice.**

**(A)** The results of HE from the other two parallel experiments in Figure 3F. Scale bar: 100 μm. **(B)** The results of IHC from the other two parallel experiments in Figure 3G. Scale bar: 100 μm.

# **Fig. S3 The level of IFN-γ and granzyme B of positive NK cells after PRV infection.**

The IFN-γ and granzyme B positive NK cells were analyzed by flow cytometry in the lung single-cell suspension from WT and *Gsdmd^-/-^* mice infected with PRV 0 h, 24 h, 48 h, 72 h and 96 h.

# **Fig. S4 The results of HE and IHC of lung tissues from WT mice and *Gsdmd^-/-^* mice infected with PRV in PBS group, anti-AsGM1 group and NK group.**

**(A)** The results of HE and IHC from the other two parallel experiments in Figure 5I. Scale bar: 100 μm.

# **Fig. S5 Flow diagram of adoptive transfer.**

**(A)** KEGG analysis of Fabp4+AMs. **(B)** The results of the expression of IL-1α from the other two parallel experiments in Figure 6B. **(C)** Process diagram of adoptive transfer of macrophages. **(D)** The clearance effect of macrophages was assessed by flow cytometry after using clodronate liposomes. **(E)** Process diagram of adoptive transfer of NK cells. **(F)** The clearance effect of NK cells was assessed by flow cytometry after using anti-AsGM1.

# **Fig. S6 GSDMD inhibitor can reduce the production of TNF-α induced by PRV infection.**

The WT mice were treated as Fig. 7. **(A)** The level of TNF-α in serum was measured by ELISA. **(B)** The level of TNF-α in lung tissues was measured by ELISA. **(C)** HE and IHC results from other two parallel experiments in Figure 7G. Scale bar: 100 μm. **, *P* < 0.01; ***, *P* < 0.001.
