# Supplementary figures and images for: Pseudorabies virus induces natural killer cell depletion by GSDMD-mediated inflammation and pyroptosis to promote infection and lung injury
Source: J Virol. 2025 Jul 24;99(8):e00415-25. doi: 10.1128/jvi.00415-25 (PMC12363163; doi:10.1128/jvi.00415-25)

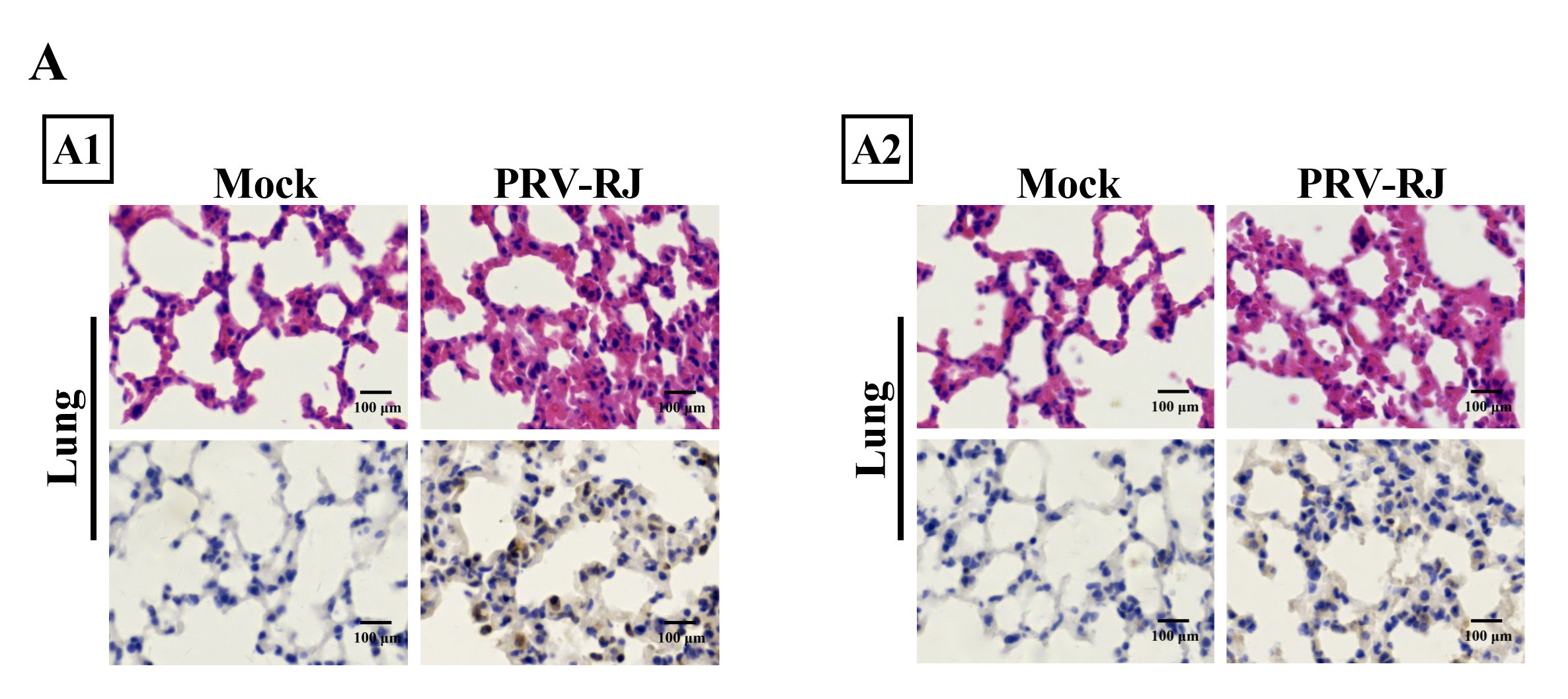

Supplement: Fig. S1 — Results of HE and IHC of mock-infected lung tissue and PRV-RJ-infected lung tissue. [file jvi.00415-25-s0002.tif]

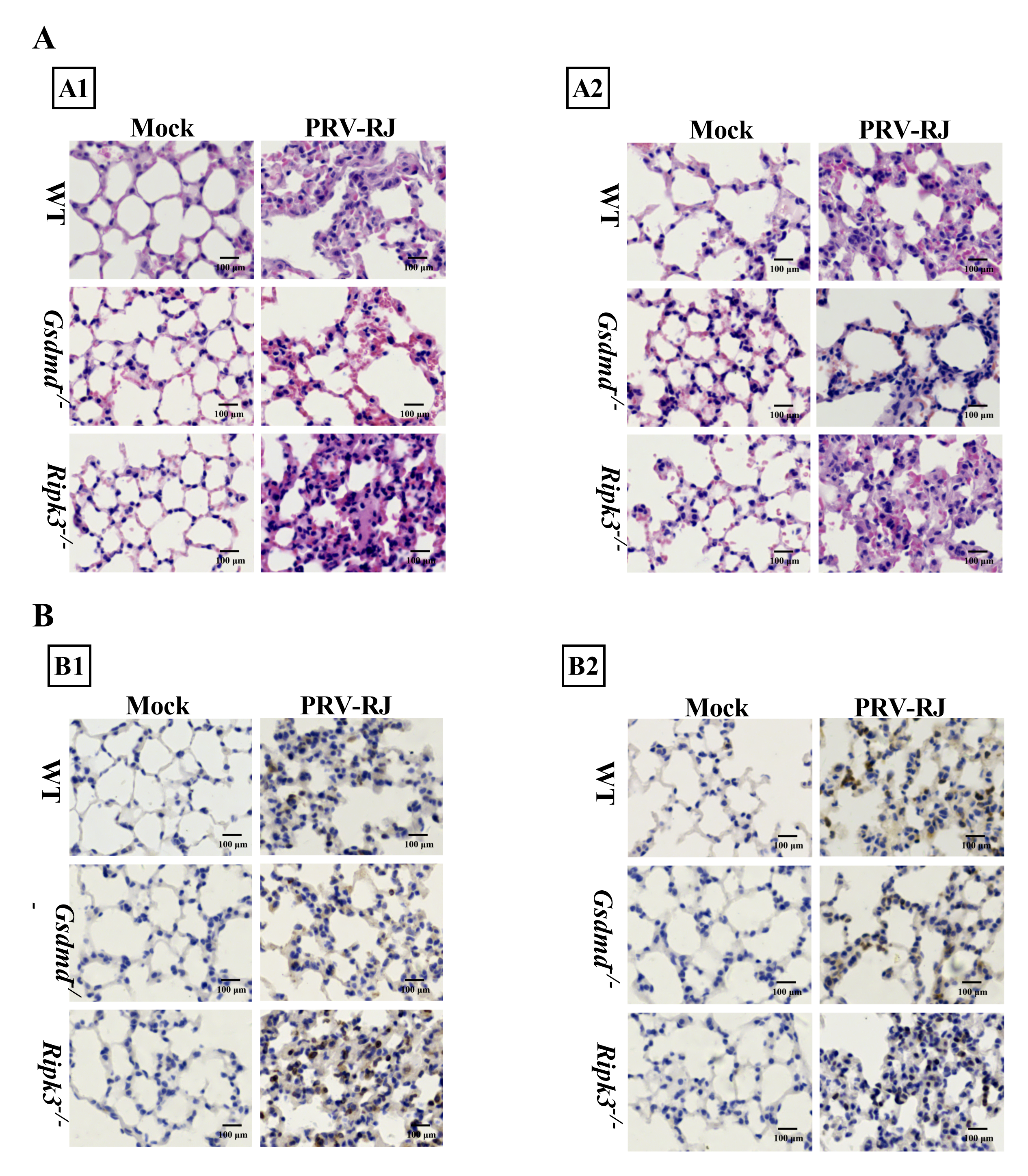

Supplement: Fig. S2 — Results of HE and IHC of mock-infected lung tissue and PRV-RJ-infected lung tissue from WT mice, Gsdmd-/- mice, and Ripk3-/- mice. [file jvi.00415-25-s0003.tif]

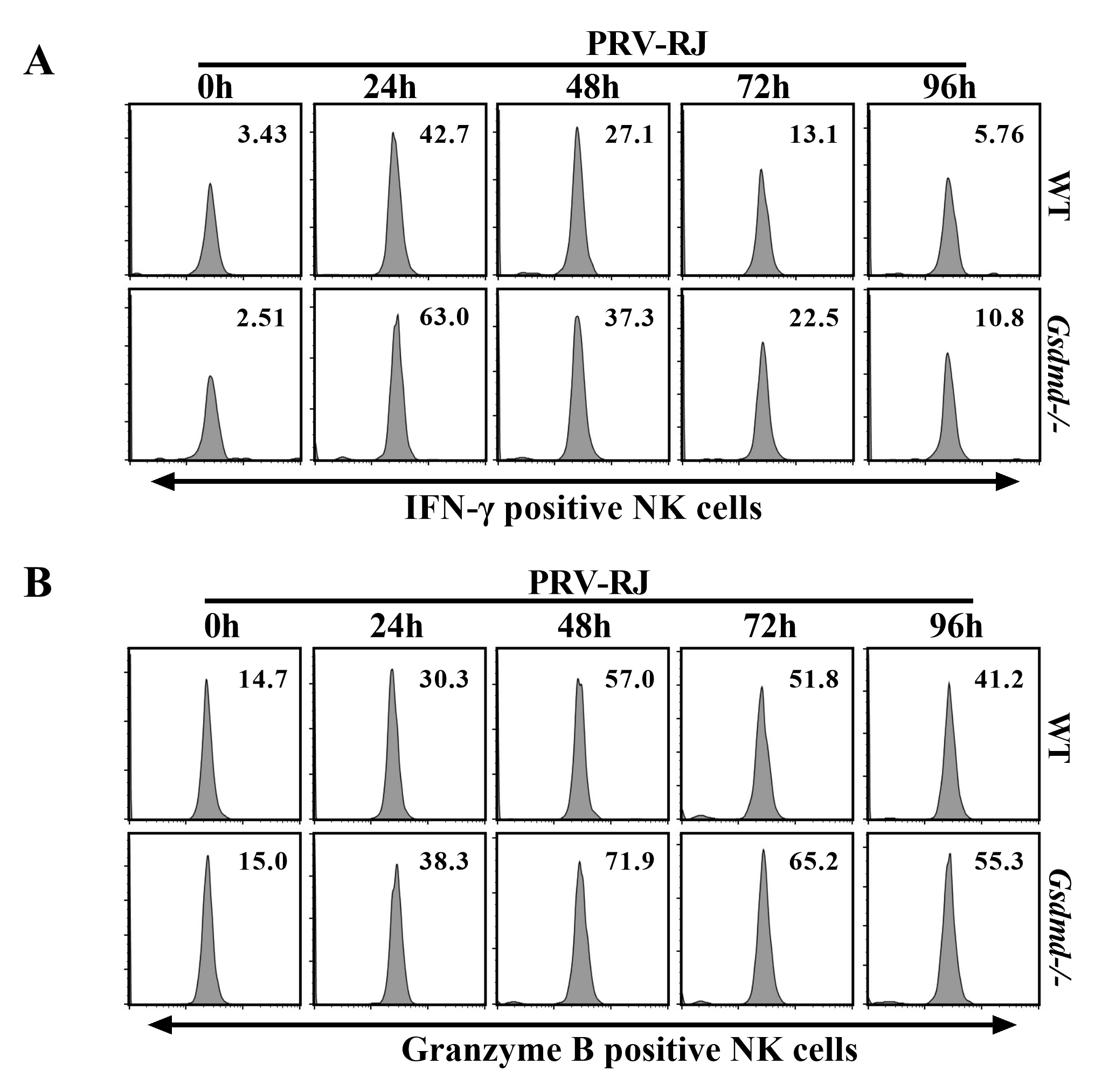

Supplement: Fig. S3 — Levels of IFN-γ and granzyme B in NK cells after PRV infection. [file jvi.00415-25-s0004.tif]

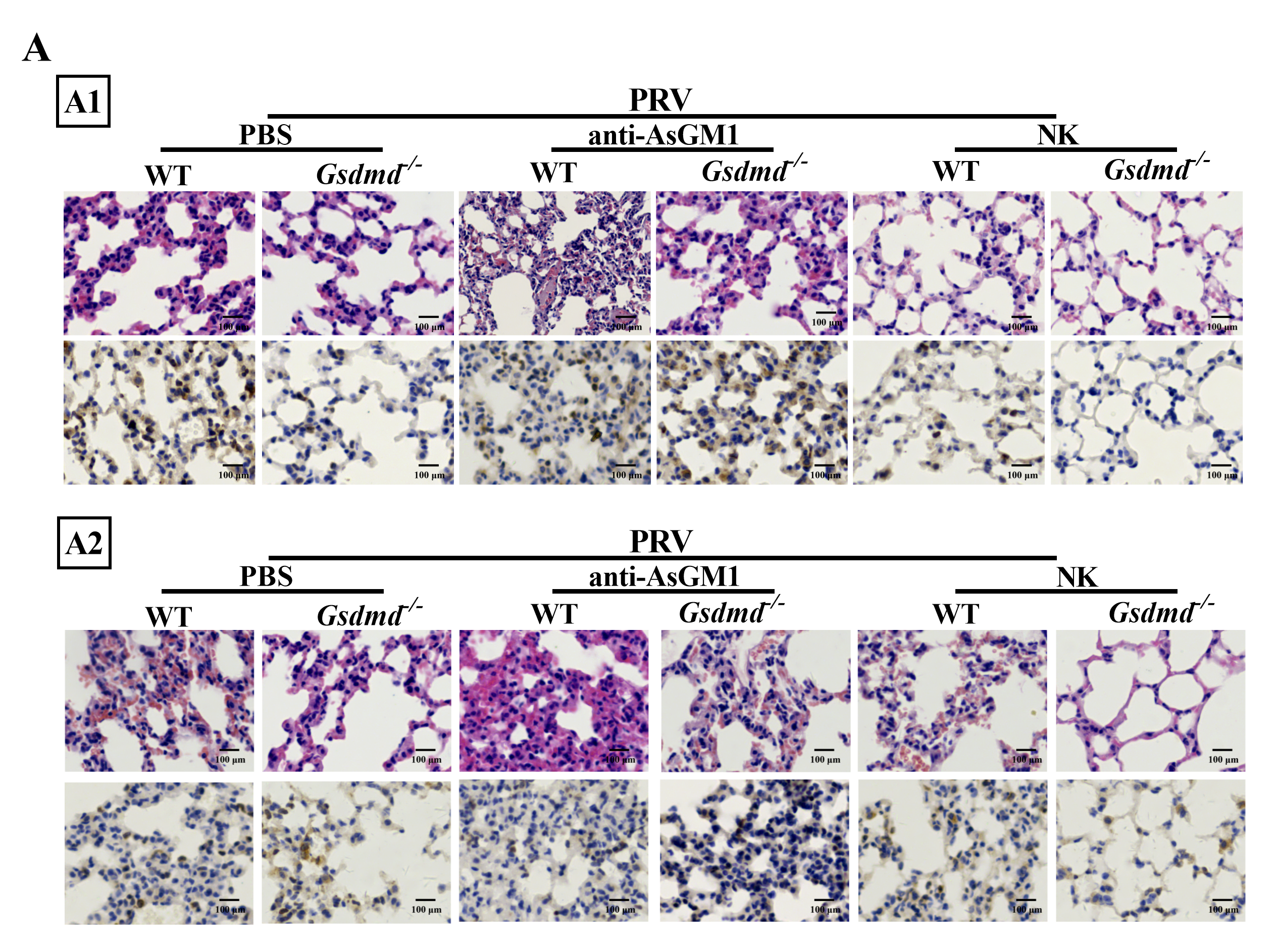

Supplement: Fig. S4 — Results of HE and IHC of lung tissues from WT mice and Gsdmd-/- mice infected with PRV in PBS group, anti-AsGM1 group, and NK group. [file jvi.00415-25-s0005.tif]

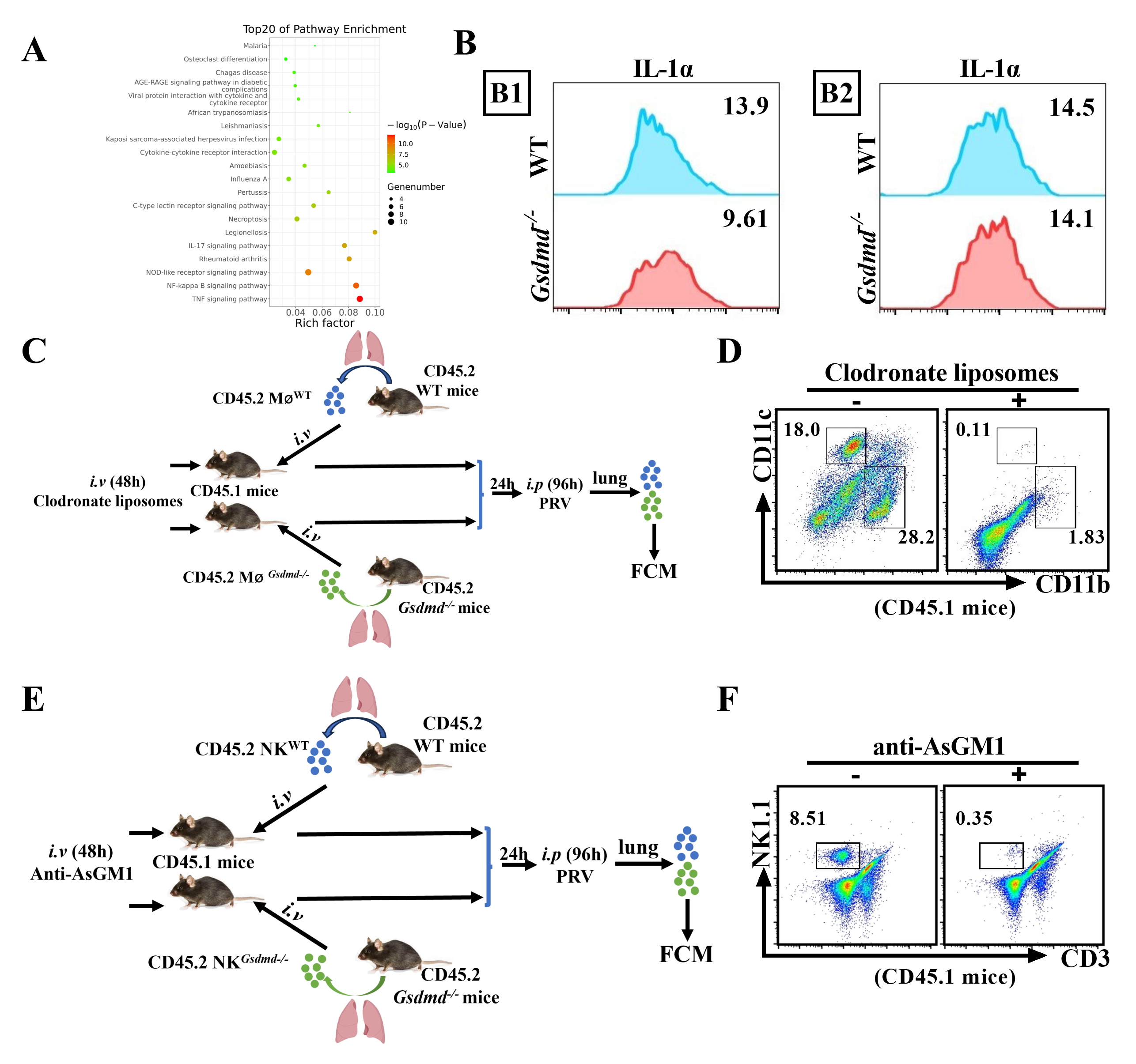

Supplement: Fig. S5 — Flow diagram of adoptive transfer. [file jvi.00415-25-s0006.tif]

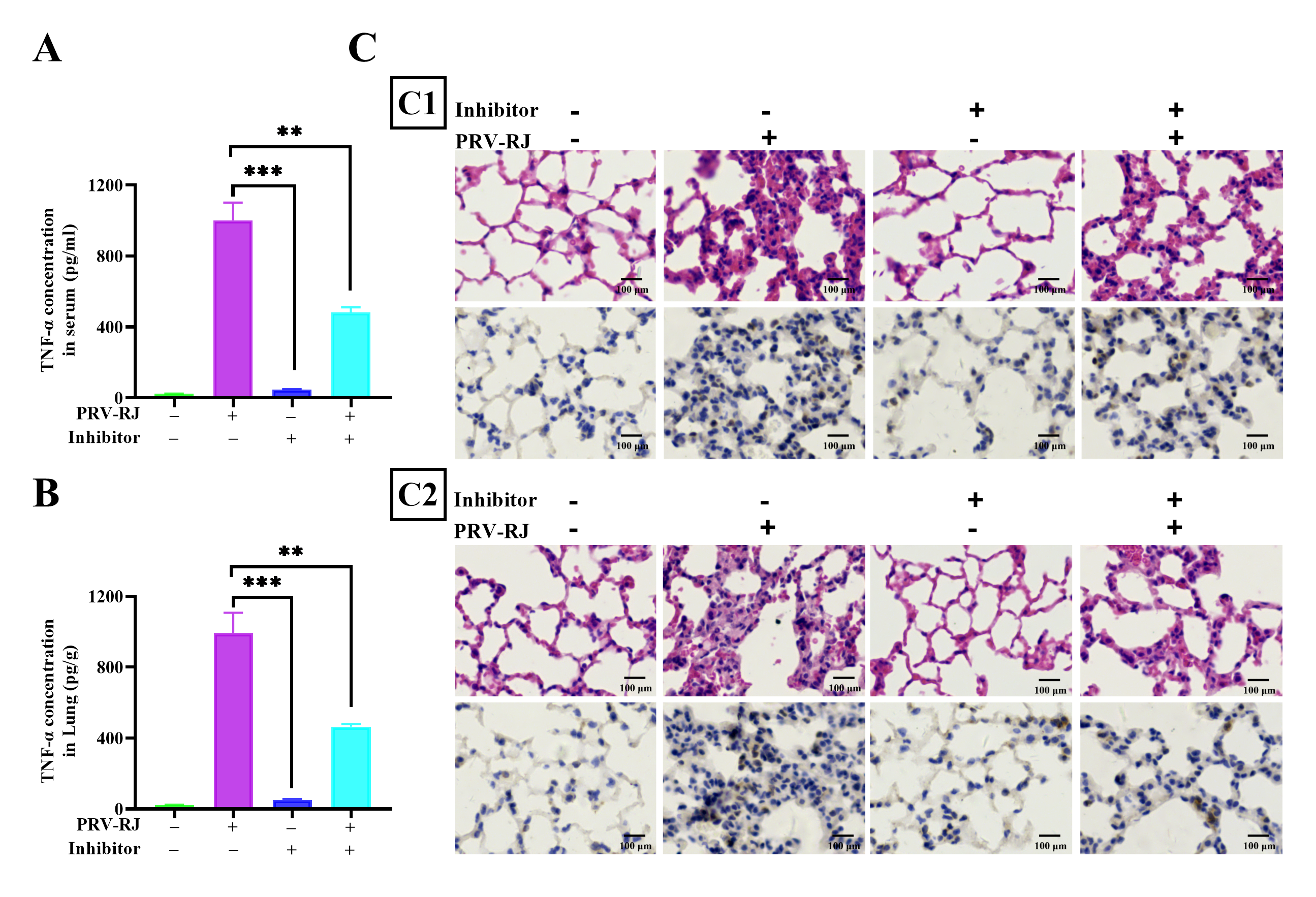

Supplement: Fig. S6 — GSDMD inhibitor can reduce the production of TNF-α induced by PRV infection. [file jvi.00415-25-s0007.tif]
