# Supplementary material for: Pseudorabies virus induces natural killer cell depletion by GSDMD-mediated inflammation and pyroptosis to promote infection and lung injury
Source: J Virol. 2025 Jul 24;99(8):e00415-25. doi: 10.1128/jvi.00415-25 (PMC12363163; doi:10.1128/jvi.00415-25)
Supplement: Supplemental results — Three parallel results of caspase 1 from primary figures. [file jvi.00415-25-s0008.docx]

1. **Three parallel results of caspase1 in Figure 1F**


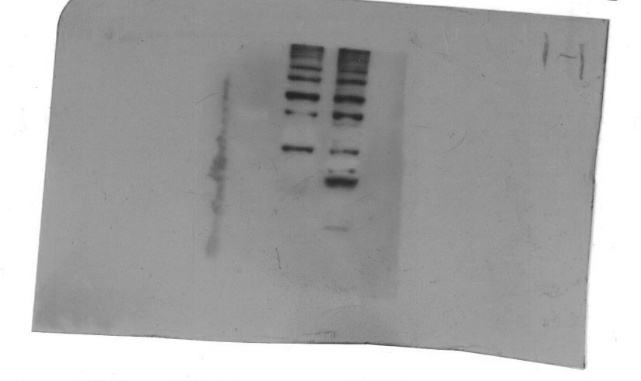

**Figure 1F-parallel result-1**


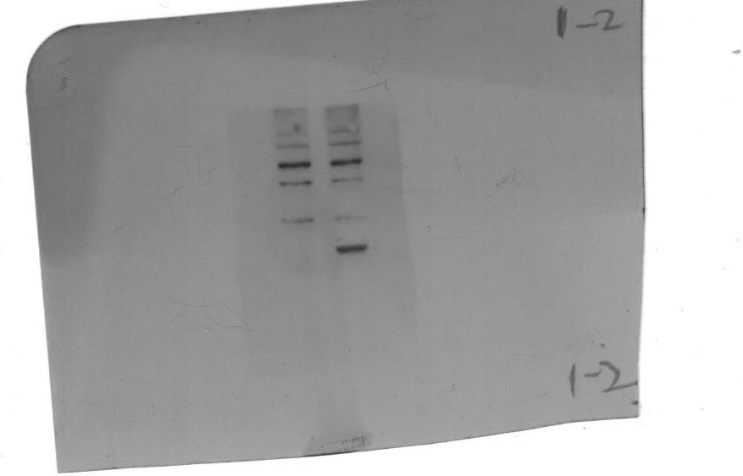


**Figure 1F-parallel result-2**


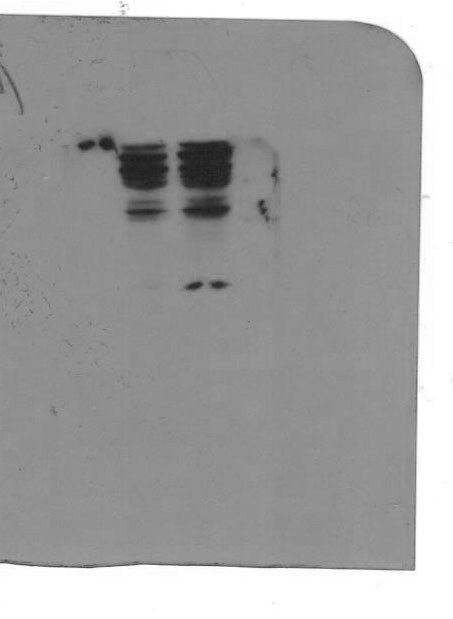


**Figure 1F-parallel result-3**

1. **Three parallel results of caspase1 in Figure 1G**


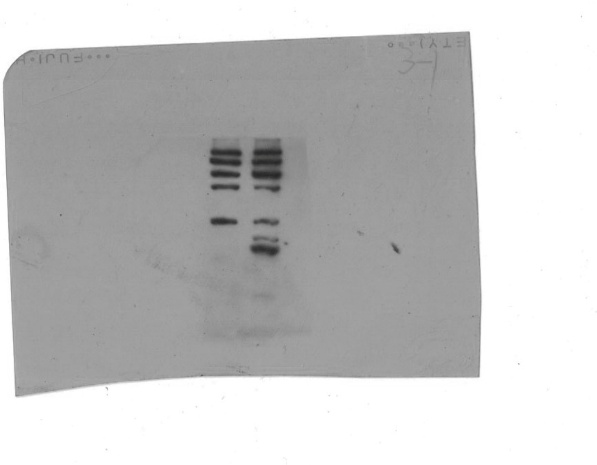

**Figure 1G-parallel result-1**


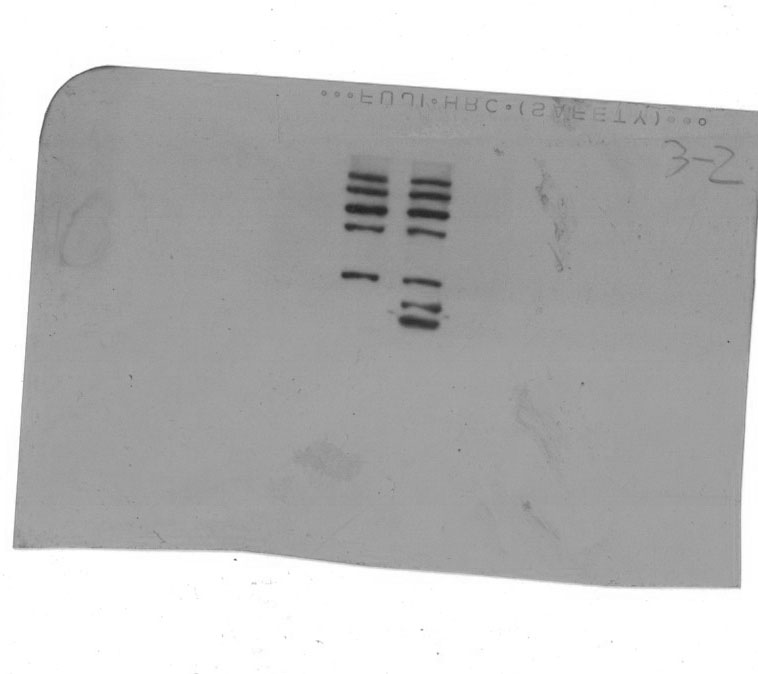


**Figure 1G-parallel result-2**


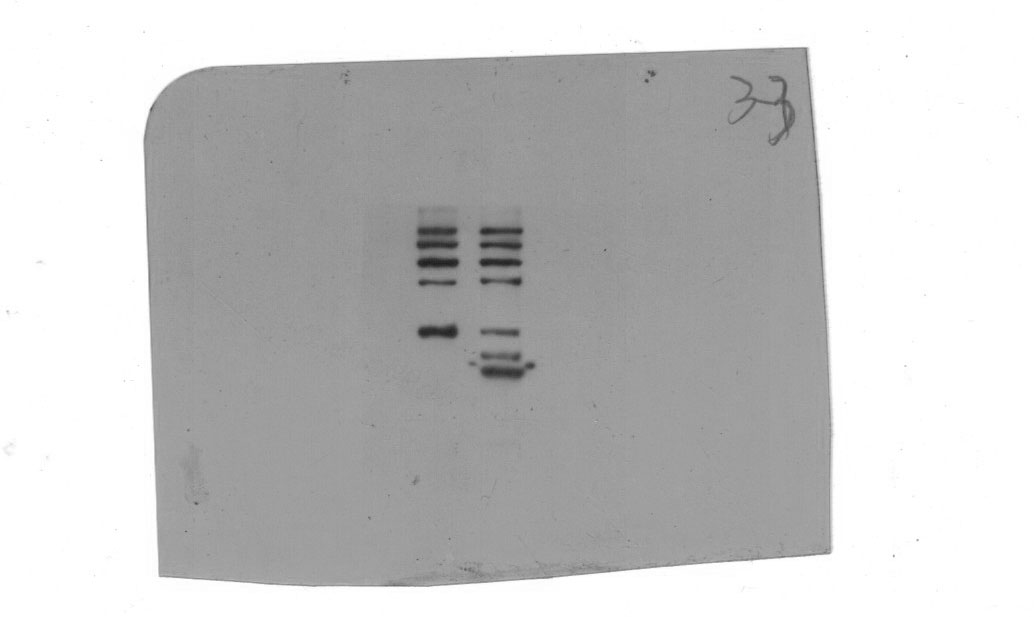


**Figure 1G-parallel result-3**

1. **Three parallel results of caspase1 in Figure 1H**


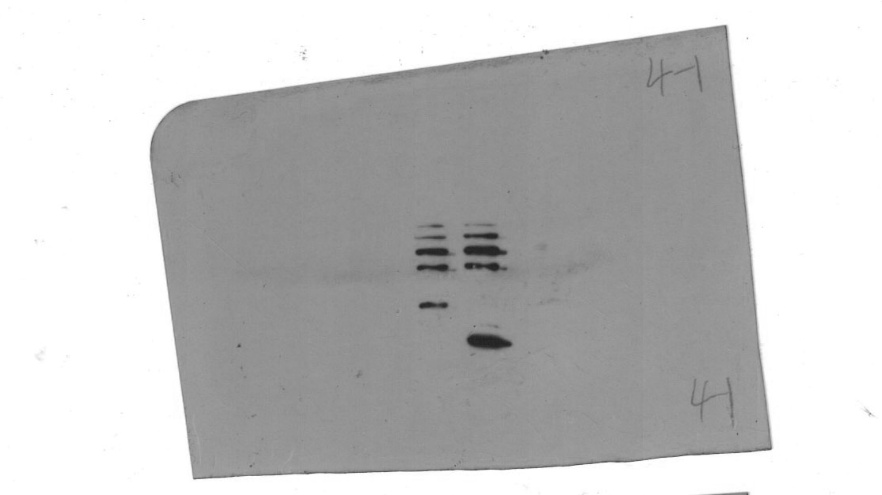

**Figure 1H-parallel result-1**


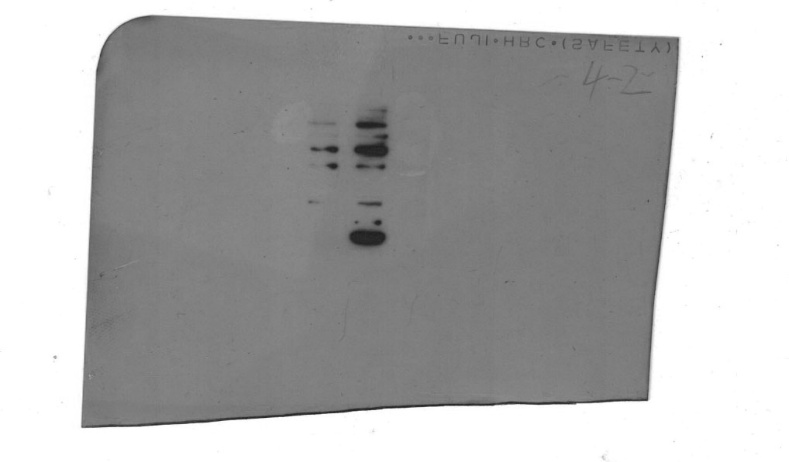


**Figure 1H-parallel result-2**


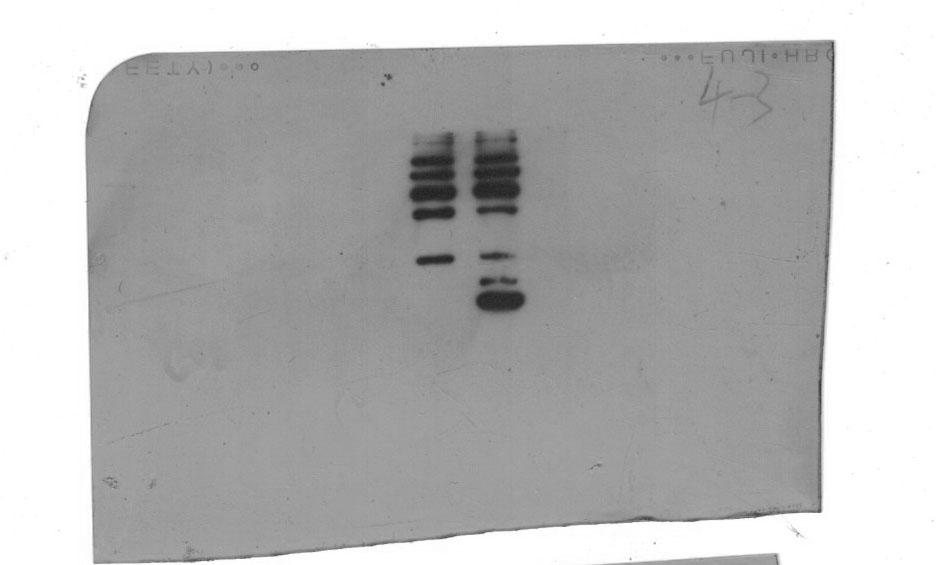


**Figure 1H-parallel result-3**

1. **Three parallel results of caspase1 in Figure 2J**


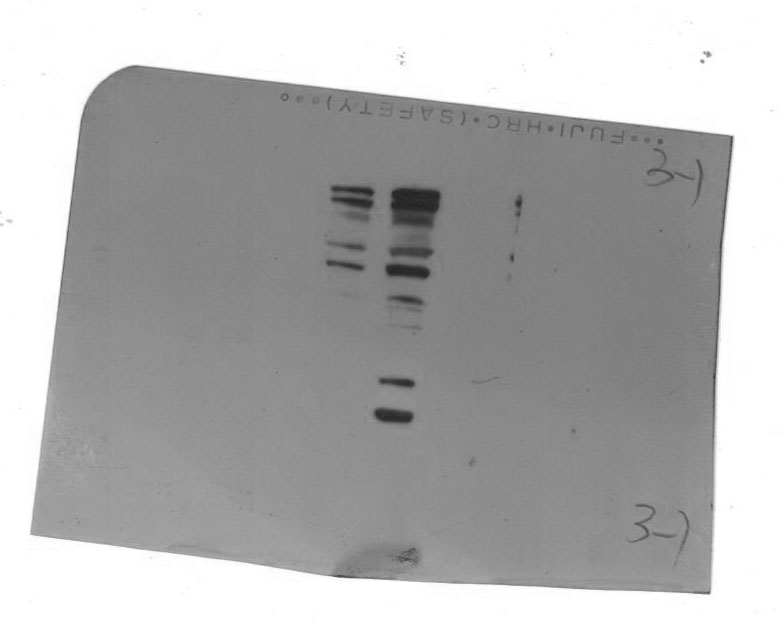

**Figure 2J-parallel result-1**


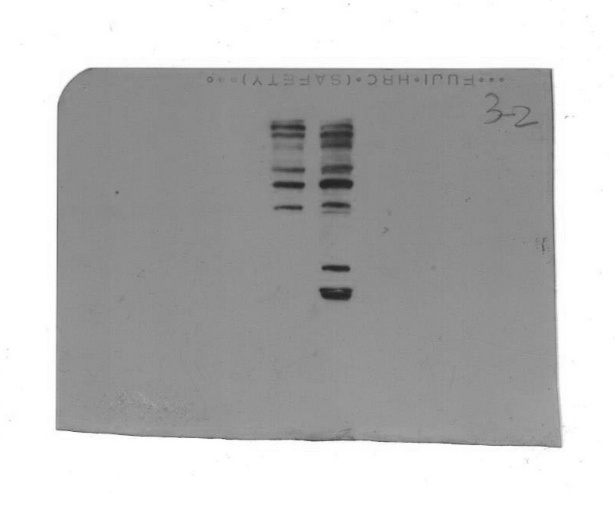


**Figure 2J-parallel result-2**


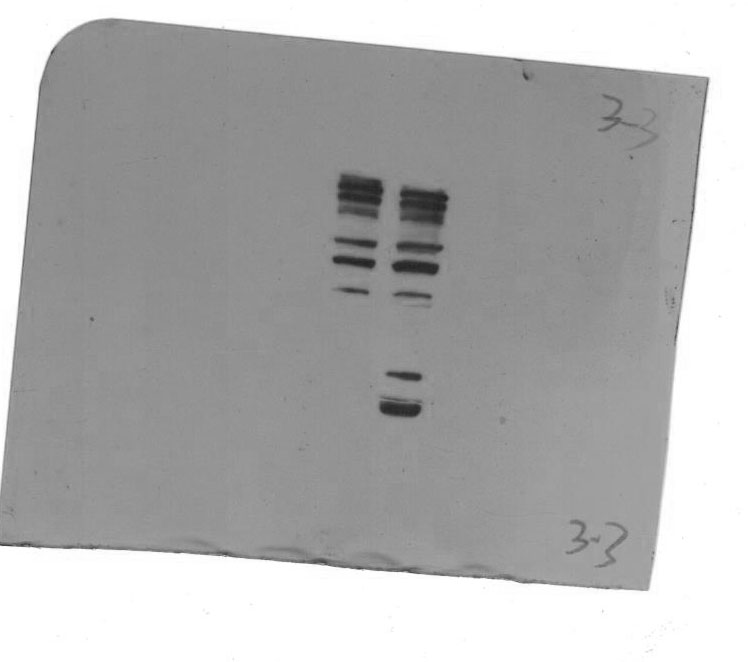


**Figure 2J-parallel result-3**

1. **Three parallel results of caspase1 in Figure 2K**


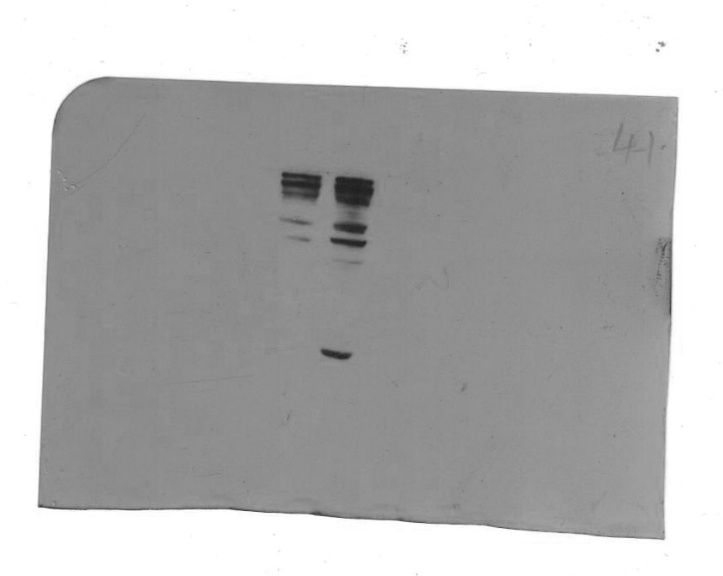

**Figure 2K-parallel result-1**


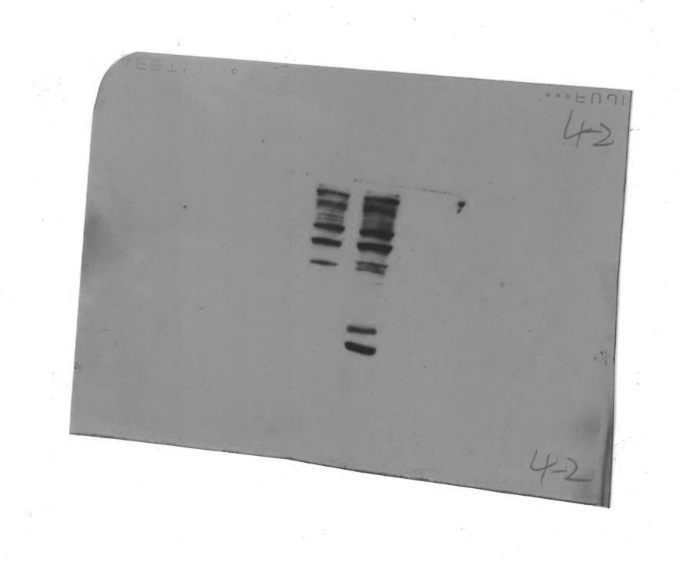


**Figure 2K-parallel result-2**


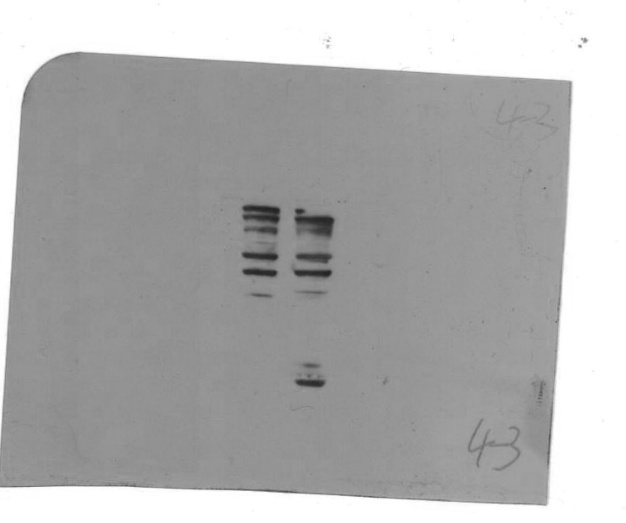


**Figure 2K-parallel result-3**

1. **Three parallel results of caspase1 in Figure 3K**


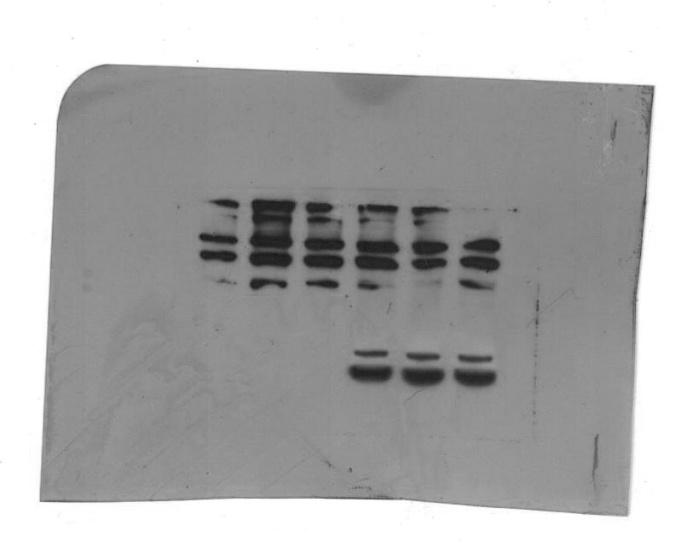

**Figure 3K-parallel result-1**


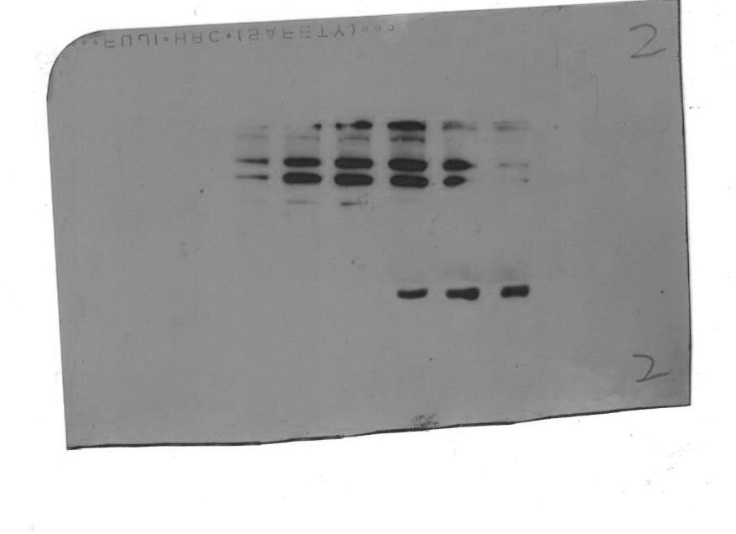


**Figure 3K-parallel result-2**


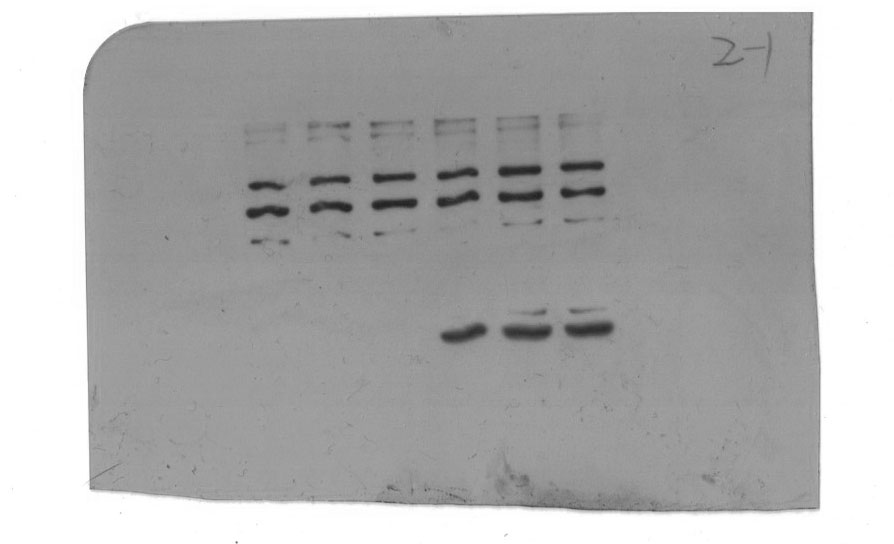


**Figure 3K-parallel result-3**

1. **Three parallel results of caspase1 in Figure 5E**


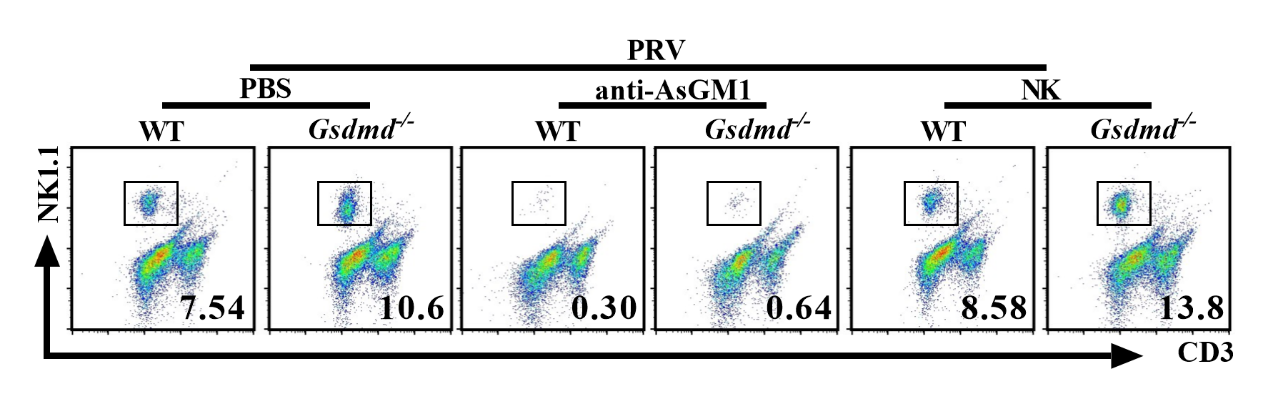

**Figure 5E-parallel result-1**


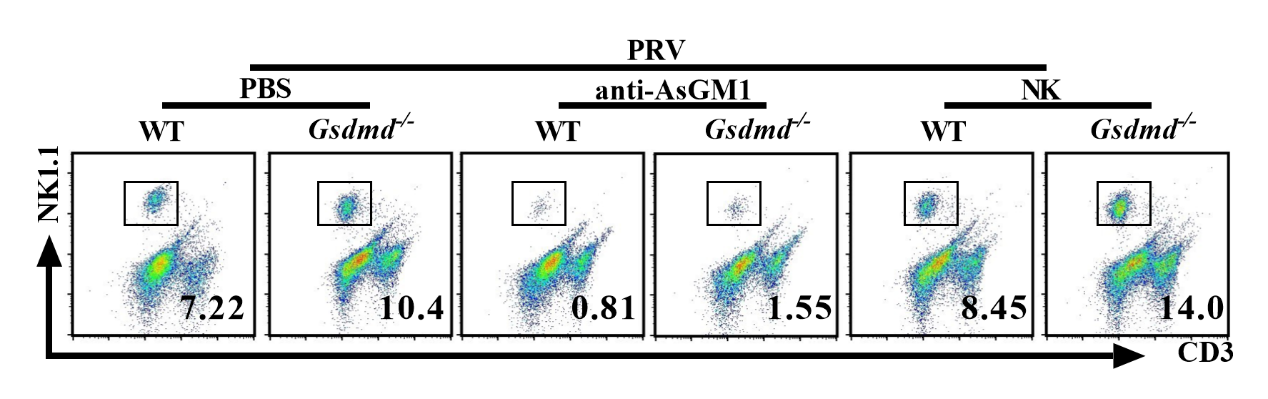


**Figure 5E-parallel result-2**


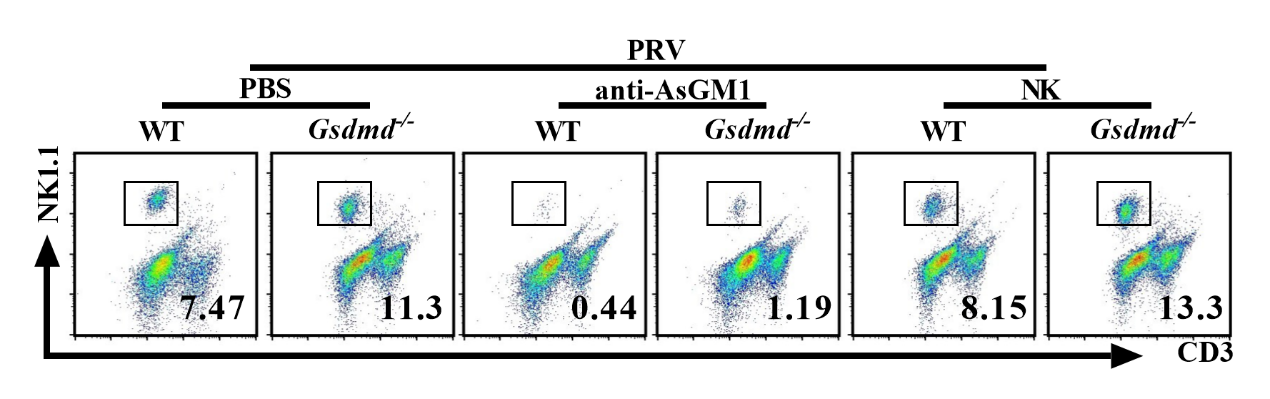


**Figure 5E-parallel result-3**

1. **Three parallel results of caspase1 in Figure 7B**

**
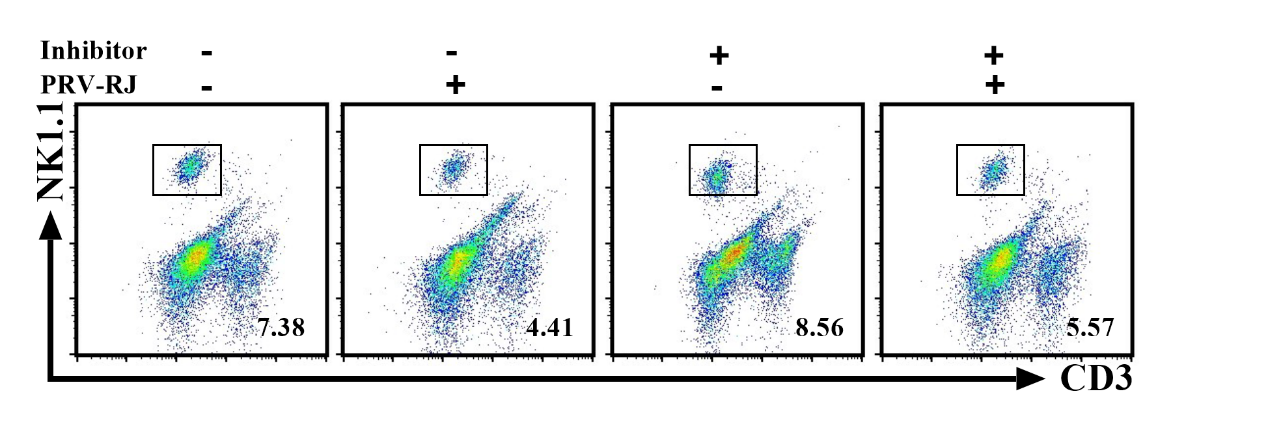
**
**Figure 7B-parallel result-1**


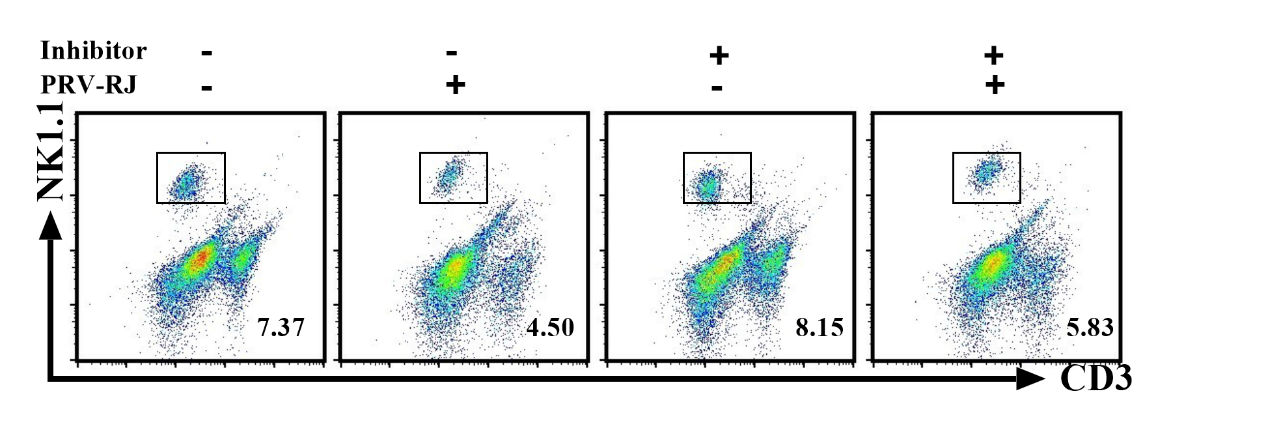


**Figure 7B-parallel result-2**


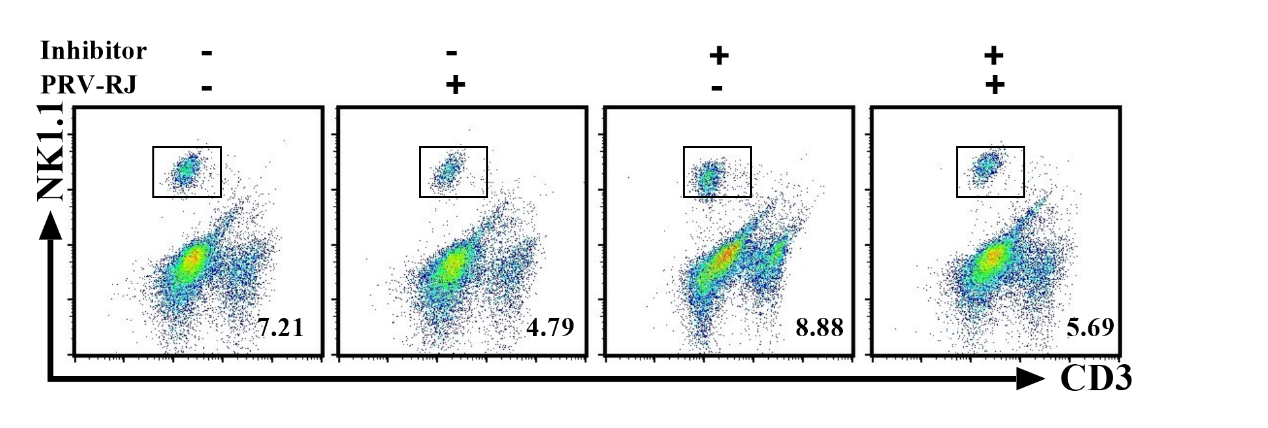


**Figure 7B-parallel result-3**
