# Supplementary material for: Pseudorabies virus induces natural killer cell depletion by GSDMD-mediated inflammation and pyroptosis to promote infection and lung injury
Source: J Virol. 2025 Jul 24;99(8):e00415-25. doi: 10.1128/jvi.00415-25 (PMC12363163; doi:10.1128/jvi.00415-25)
Supplement: Supplemental tables — Single cell sequencing data. [file jvi.00415-25-s0009.docx]

**Supplement Table 1 Statistics of scRNA-seq**

| **Sample** | **GSDMD** | **WT** |
| --- | --- | --- |
| **Estimated Number of Cells** | 6,071 | 7,204 |
| **Mean Reads per Cell** | 56,972 | 57,058 |
| **Median Genes per Cell** | 1,608 | 1,724 |
| **Number of Reads** | 345,875,607 | 411,043,699 |
| **Valid Barcodes** | 97.8% | 97.8% |
| **Sequencing Saturation** | 71.7% | 69.3% |
| **Q30 Bases in Barcode** | 95.7% | 95.7% |
| **Q30 Bases in RNA Read** | 92.0% | 92.0% |
| **Q30 Bases in UMI** | 94.7% | 95.1% |
| **Reads Mapped to Genome** | 93.7% | 93.6% |

**Supplement Table 2 Canonical markers used to annotate each cell type cluster**

| **Cluster** | **GSDMD** | **WT** | **Sum** | **Cell type** | **Marker genes** |
| --- | --- | --- | --- | --- | --- |
| 0 | 627 | 916 | 1543 | AlveolarMacrophages | Mcemp1, Macro, Fabp4, Pparg |
| 1 | 517 | 785 | 1302 | Bcell | Cd19, Cd79a, Cd22, Msa41 |
| 2 | 384 | 608 | 992 | Tcell | Ptprc, Cd3d, Cd3e, Cd3g, Cd4, Cd8a |
| 3 | 396 | 556 | 952 | Neutrophil | S100a9, Csf3r, G0s2 |
| 4 | 511 | 355 | 866 | NK | Ncr1, Gzma, Nkg7, Klre1 |
| 5 | 211 | 387 | 598 | Monocyte-DC | Vcan, Cd74, Cd209a, Fscn1 |
| 6 | 273 | 262 | 535 | Fibroblast | Dcn, Col1a1, Sparc |
| 7 | 243 | 284 | 527 | Tcell | Ptprc, Cd3d, Cd3e, Cd3g, Cd4, Cd8a |
| 8 | 151 | 260 | 411 | Macrophages | C1qa, C1qb, Apoe |
| 9 | 149 | 252 | 401 | Endothelial | Pecam1, Cldn5, Vwf |
| 10 | 115 | 168 | 283 | Monocyte-DC | Vcan, Cd74, Cd209a, Fscn1 |
| 11 | 143 | 119 | 262 | Endothelial | Pecam1, Cldn5, Vwf |
| 12 | 113 | 118 | 231 | mix-CyclingCell | Mki67, Stmn1, Top2a |
| 13 | 53 | 92 | 145 | Epithelial | Epcam, Krt8, Krt18 |
| 14 | 88 | 48 | 136 | Endothelial | Pecam1, Cldn5, Vwf |
| 15 | 78 | 57 | 135 | AlveolarMacrophages | Mcemp1, Macro, Fabp4, Pparg |
| 16 | 52 | 75 | 127 | AlveolarMacrophages | Mcemp1, Macro, Fabp4, Pparg |
| 17 | 49 | 64 | 113 | Neutrophil | S100a9, Csf3r, G0s2 |
| 18 | 60 | 43 | 103 | Monocyte-DC | Vcan, Cd74, Cd209a, Fscn1 |
| 19 | 42 | 50 | 92 | Myofibroblast | Dcn, Col1a1, Sparc, Acta2 |
| 20 | 19 | 41 | 60 | Plasma | Cd79a, Tnfrsf17, Jchain, Lgha |
| 21 | 25 | 43 | 85 | AlveolarMacrophages | Mcemp1, Macro, Fabp4, Pparg |
| sum | 4299 | 5573 | 9872 |  |  |
