# Supplementary material for: Pseudorabies virus induces natural killer cell depletion by GSDMD-mediated inflammation and pyroptosis to promote infection and lung injury
Source: J Virol. 2025 Jul 24;99(8):e00415-25. doi: 10.1128/jvi.00415-25 (PMC12363163; doi:10.1128/jvi.00415-25)
Supplement: Supplemental table legends — Legends for Tables S1 and S2. [file jvi.00415-25-s0010.docx]

# **Table S1 Statistics of scRNA-seq from lung single-cell suspension in PRV-infected WT mice and PRV-infected *Gsdmd^-/-^* mice after infected 48 h.**

# **Table S2 Canonical markers used to annotate each cell type cluster from lung single-cell suspension in PRV-infected WT mice and PRV-infected *Gsdmd^-/-^* mice after infected 48 h.**
